# Red Phosphorus Potassium‐Ion Battery Anodes

**DOI:** 10.1002/advs.201801354

**Published:** 2019-02-28

**Authors:** Wei‐Chung Chang, Jen‐Hsuan Wu, Kuan‐Ting Chen, Hsing‐Yu Tuan

**Affiliations:** ^1^ Department of Chemical Engineering National Tsing Hua University 101, Section 2, Kuang‐Fu Road Hsinchu Taiwan 30013 Republic of China

**Keywords:** P—C bonding, PIB anodes, red phosphorus, wet‐ball milling process

## Abstract

Phosphorus (P) possesses the highest theoretical specific capacity (865 mA h g^−1^) among all the elements for potassium‐ion battery (PIB) anodes. Although Red P (RP) has intrinsic advantages over its allotropes, including low cost and nontoxicity, and simpler preparation, it is yet unknown to effectively activate it into a high‐performance PIB anode. Here, high‐performance RP PIB anodes are reported. Two important factors are found to facilitate RP react with K‐ions reversibly: i) nanoscale RP particles are dispersed evenly in a conductive carbon matrix composed of multiwall carbon nanotubes and Ketjen black that provide an efficient electrical pathway and a tough scaffold. ii) The results of X‐ray photoelectron spectroscopy spectrum and the electrochemical performance perhaps show that no P—C bond formation is beneficial to allow K‐ions to react with RP effectively. As a result, the RP/C electrodes deliver a reversible specific capacity of ≈750 mA h g^−1^ and exhibit a high‐rate capability (≈300 mA h g^−1^ at 1000 mA g^−1^). RP/C full cells using potassium manganese hexacyanoferrate as cathode show a long cycling life (680 cycles) at a current density of 1000 mA g^−1^, in addition, a pouch‐type battery is built to demonstrate practical applications.

The explosive growth of electric vehicles and large‐scale stationary electrical energy storage dramatically increase the requirements of reversible batteries as energy supply. Lithium‐ion batteries (LIBs) provide high energy density for these applications, however, the possible extraordinary demands (around three times higher than today's demand^1^) accelerate the consumption of the lithium (Li) miner.^2^ Potassium (K) is a Li alternative owing to the following advantages: i) the abundant reserves of potassium (2.09 wt%) in the Earth's crust—1000 times more common than that of Li (0.0017 wt%).^3^ ii) The standard reduction potential of K^+^/K is lower than Li^+^/Li in a nonaqueous electrolyte, indicating that the average working voltage of K‐ion batteries might be similar to that of the LIBs.^4^ iii) The Stokes radius of K‐ions (3.6 Å in polycarbonate (PC)) is the smallest as compared to that of Li‐ions (4.8 Å in PC) and Na‐ions (4.6 Å in PC), which may have higher mobility and diffusion kinetic between the electrolyte and electrode.[[qv: 4a,c]] Several cathode^5^ and anode materials[[qv: 3b,6]] have been investigated for K‐ion batteries, however, high capacity anode materials such as P (>800 mA h g^−1^)[[qv: 3b,6a,7]] and Sb (660 mA h g^−1^)^8^ are rarely reported.

Phosphorus (P) possesses the theoretical highest specific capacity (865 mA h g^−1^) among all the elements for potassium‐ion battery (PIB) anodes. For instance, Zhang reported P‐based material Sn_3_P_4_ for PIB anode that shows a reversible capacity of 385 mA h g^−1^ at 50 mA g^−1^.[[qv: 3b]] Thereafter, Sn_4_P_3_ particles embedded into the N‐doped carbon fibers that further improve the electrochemical performance.[[qv: 7b]] On the other hand, black P was demonstrated as a high capacity active material for PIB anode via the formation of KP alloy, delivering a high specific capacity that is close to the theoretical capacity of 865 mA h g^−1^.[[qv: 6a]] Recently, RP‐C composites prepared via a vaporization–condensation process shows a specific capacity of around 700 mA h g^−1^,[[qv: 7d]] however, the process is quite complicated since it has to be carried out at high temperature (600 °C) for over 30 h.

RP is an easily manufacturing and low cost material relative to its P allotropes, nevertheless, how to effectively use RP as an anode material for PIBs is unclear. Herein, we report high‐performance red phosphorus (RP) PIB anodes. We develop a one‐pot wet‐ball milling (WBM) approach to prepare the electrode slurry composed of RP, multi‐wall carbon nanotube (MWCNT), Ketjen black (KB), and sodium carboxymethyl cellulose (NaCMC). The RP/MWCNT/KB (RP/C) electrode evaluated in the potassium half‐cells showed a high reversible capacity of 750 mA h g^−1^ as well as a high‐rate capability (≈300 mA h g^−1^ at the current density of 1000 mA g^−1^). A ball milling process can reduce commercial RP size down to less than 500 nm and obtain composites fully covered with MWCNT and KB that enhance electric conductivity and structure strength. Moreover, to activate RP as promising K‐ion anodes, we found that it may need to avoid the formation of P—C bonds in RP‐C composites, which is possibly unfavorable to the alloying reactions between RP and K‐ions. The structural configuration of an RP/C electrode is shown in **Scheme** **1**. In the end, potassium‐ion full cells composed of RP/C as anode and potassium manganese hexacyanoferrate (KMnHCF) as cathode were evaluated. The full cells exhibited a high working voltage (≈3.4 V), long‐term cycling life (680 cycles) and large specific energy density (193 Wh kg^−1^), demonstrating that the RP/C is a promising candidate as an anode material for PIB.

**Scheme 1:**
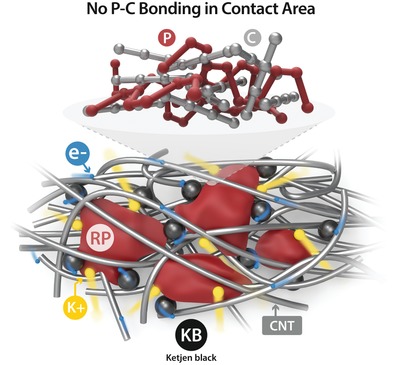
Schematic illustration of structural configuration of activated RP/C‐based PIB electrode without the formation of P—C bonds.

RP/C electrodes were fabricated by mixing commercial RP, MWCNT, KB, NaCMC, and water via a one‐pot WBM process in a stainless steel jar. A homogenous slurry with deep red color formed after a 12 h milling process (**Figure** **1**a). Figure 1b,c shows the scanning electron microscopy (SEM) images of the surface of RP‐based electrode. The average size of an RP particle is around 200–500 nm in diameter and is distributed uniformly in the conducting framework built by MWCNT and KB, as shown in Figure S1a,b (Supporting Information). In the composites, most of the MWCNT and KB still remained original morphologies (Figure S2, Supporting Information) and blend with NaCMC to form continuous electrical pathways as well as a tough scaffold. Via this structure, nanoscale RP could be electrochemically activated. Figure 1d,g shows the SEM images of RP/C powder dried from the slurry. Figure 1e,f,h,i presents the energy dispersive spectroscopy (EDS) mapping images corresponding to Figure 1d,g, respectively, revealing that nanoscale RP particles are distributed even in the carbon matrix. Transmission electron microscopy and EDS mapping images further show that some RP particles with the sizes of <50 nm were mixed into carbon composed of deformed KB and MWCNTs (Figure S1c–g, Supporting Information). The X‐ray diffraction (XRD) pattern recorded from the RP/C dry powder is displayed in Figure 1j. The position of three broadened diffraction peaks at 13°–16°, 25°–38°, and 47°–65° are in accordance with the XRD pattern of commercial RP.^9^ The weaker diffraction peaks of RP in RP/C relative to commercial RP indicates some P—P bonds broken in the course of WBM. A small peak at around 25° is the characteristic (002) reflection of MWCNT and KB.^10^ The Raman spectrum of RP/C dry powder (Figure 1k) is dominated by three bands between 300 and 500 cm^−1^, which is consistent with the spectrum of commercial RP. In the region of 1200–1800 cm^−1^, the presence of the two peaks located at around 1350 and 1600 cm^−1^ corresponding to the D band and G band, respectively, is the typical feature of the MWCNT and KB.^11^ Figure 1l shows P2p spectrum of X‐ray photoelectron spectroscopy (XPS) measurement fitted to three peaks at around 129.7, 130.6, and 133.5 eV. The first two peaks are assigned to the P—P bond,^12^ while the last peak is assigned to the P—O bond.[[qv: 9b,12a]] The formation of P—O bonds was due to the WBM process that was operated in atmosphere, leading to the oxidation of RP surface.

**Figure 1:**
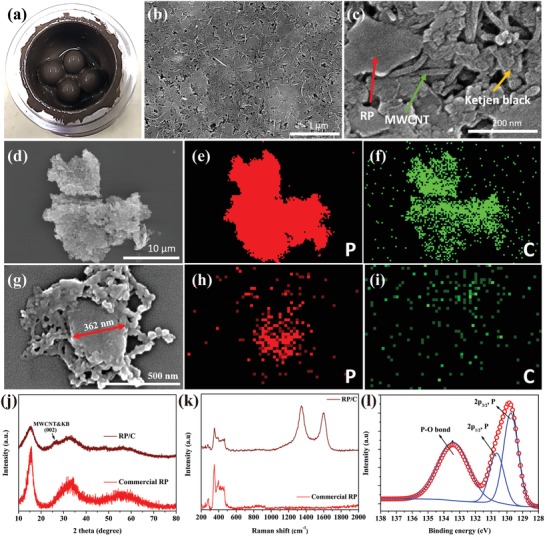
Morphology and characterization of RP/C. a) Image of RP/C after a WBM process in a stainless steel jar. b) SEM image of the surface of RP/C electrode. c) High‐magnification SEM image of RP/C electrode. The red, green, and yellow arrows figure out the RP particles, MWCNT, and KB, respectively. d) Low‐magnification SEM images. g) High‐magnification SEM images. e,f) and h,i) EDS mapping images corresponding to (d) and (g), respectively. The red and green dots represent phosphorus and carbon, respectively. j) XRD patterns of RP/C and commercial RP. The black arrow figures out the characteristic reflection of MWCNT and KB. k) Raman spectra of RP/C and commercial RP. l) P2_p_ spectrum of XPS of RP/C.

The electrochemical performance of RP/C electrode was evaluated by galvanostatic discharge/charge measurements in the range of 0.01–2.5 V using coin‐type CR2032 half‐cells composed of a handmade potassium foil as a counter electrode. The electrolyte solution in the half‐cells was 1 m potassium bis(trifluoromethanesulfonyl)imide (KTFSI) in ethylene carbonate/diethyl carbonate (EC/DEC, 1:1 vol%). **Figure** **2**a shows the cyclic voltammetry (CV) plot of the RP/C electrode. A broad peak which only appears at the first cathodic cycle located in the region of 0.7–1.1 V could be attributed to the irreversible reactions of MWCNT/KB with potassium ions (see the voltage profile of MWCNT/KB electrode in Figure S3 in the Supporting Information) and the formation of the solid electrolyte interface (SEI). In the following cycle, RP/C electrode maintained consistent current–voltage curves in both cathodic and anodic scans. For the cathodic cycle, an apparent peak ranging from 0.5 to 0.01 V is the electrochemical alloying process of RP with potassium ion, forming continuously from K*_x_*P to KP. The ex situ XRD pattern further confirms that KP is the only phase in the termination of the potassiation process, as shown in Figure S4 (Supporting Information). This result is accordant with the literature reported by Sultana et al.,[[qv: 6a]] indicating that the theoretical specific capacity of RP is 865 mA h g^−1^ based on the KP alloy phase (the calculation of theoretical capacity of KP alloy is shown in the Supporting Information). For the anodic cycle, besides one peak appeared apparently at around 0.7 V, there is a broad peak in the region of 1.5–2.5 V, revealing a stepwise depotassiation process that extracted the potassium from KP alloy. Figure 2b–g represents the electrochemical performance of RP/C half‐cells. The calculation of the specific capacity is based on the mass of RP owing to the significantly lower specific capacity (<50 mA h g^−1^) contributed by MWCNT and KB, which is small enough to be negligible (Figure S3 in the Supporting Information shows the electrochemical performance of MWCNT/KB in the half‐cell). Figure 2b,c shows that RP/C as a K‐ion storage material exhibited a specific discharge capacity of 914.8 mA h g^−1^ through the first potassiation process at the current density of 25 mA g^−1^, whereas bulk RP directly mixed with MWCNT, KB, NaCMC showed a deactivated behavior with the specific capacity of 15 mA h g^−1^. After finishing the first depotassiation process, the RP/C electrode delivered a charge capacity of 624.5 mA h g^−1^, corresponding to a Coulombic efficiency of 68.26%. In the following cycles, RP/C displayed an average reversible charge capacity exceeded 750 mA h g^−1^.

**Figure 2:**
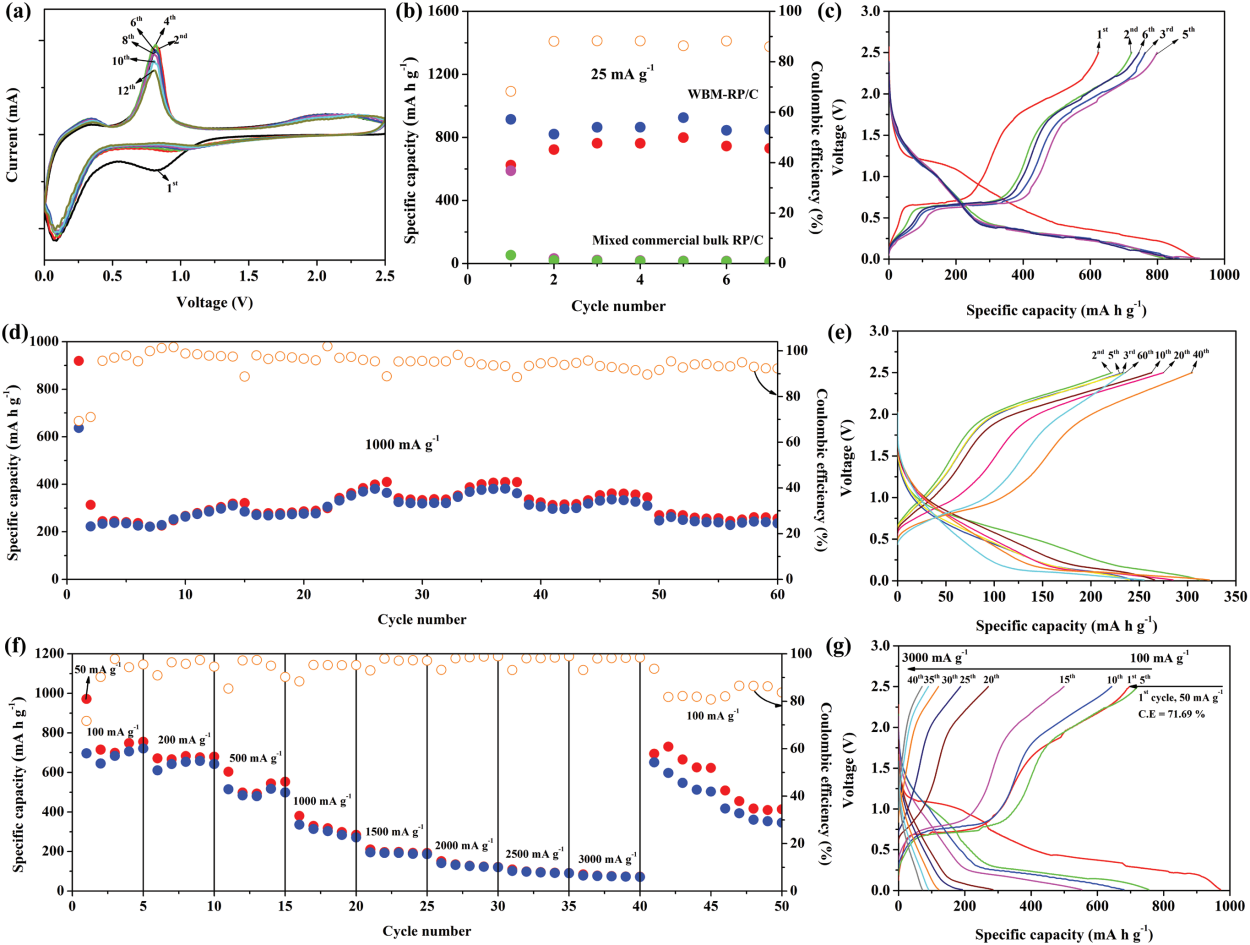
Electrochemical performance of RP/C electrode: a) cyclic voltammetry evaluation of RP/C electrode at a scan rate of 0.1 mV s^−1^. b) Cycling performance of RP/C electrode prepared from different mixing methods at the current density of 25 mA g^−1^ and c) the corresponding voltage profile. d) The cycling performance of RP/C electrode at a current density of 1000 mA g^−1^ over 60 cycles and e) the corresponding voltage profile. f) The rate performance of RP/C electrode at various current densities from 100 to 3000 mA g^−1^. The first cycle was operated at the current density of 50 mA g^−1^ and g) the corresponding voltage profile.

RP/C cycles at various discharge/charge current densities were carried out (Figure 2d,f). Their corresponding voltage profiles are shown in Figure 2e,g, respectively. RP/C exhibits a stable cycling performance of 60 cycles at a current density of 1000 mA g^−1^, delivering an average reversible specific capacity of around 300 mA h g^−1^. Besides, for the evaluation of the rate capability, the recorded charge capacities were 721, 643, 499, 270, 187, 121, 90, and 71 mA h g^−1^ at the current density of 100, 200, 500, 1000, 1500, 2000, 2500, and 3000 mA g^−1^, respectively. When the current density was returned to 100 mA g^−1^, the RP/C electrode still retained a high charge capacity of 651 mA h g^−1^, corresponding to a retention of 90.3% with respect to the fifth cycle. However, the specific capacity of RP/C began to occur continuous decay from 42th cycle until 50th cycle that remains only 413 mA h g^−1^. To investigate the possible failure cause, a half‐cell corresponding to Figure 2f was disassembled (Figure S5, Supporting Information). We found that the separator was covered with yellow powder, revealing that the electrolyte was almost dry out. The gradually decreasing electrolyte influences the diffusion of potassium ion, leading to rapid capacity fade. We speculate that there are some side reactions between potassium metal, RP/C, and electrolyte during the electrochemical evaluation. As shown in Figure S6 (Supporting Information), even in the glove box under an argon atmosphere, the potassium metal soaked in the electrolyte resulted in a color change of the electrolyte from the transparency to the yellow after few days. This result indicates that the side reaction occurred between the potassium metal and electrolyte; moreover, the side reactions might be intensified during the electrochemical evaluation. We have tried to stabilize the electrochemical performance of RP/C half‐cells by adding fluoroethylene carbonate (FEC, 5 vol%) as an additive. Although FEC has been demonstrated effective on stabilizing electrode performance on both LIBs and sodium‐ion batteries, FEC was functionless for the RP/C electrode. The electrode with FEC‐included electrolyte only exhibited the specific capacity of <3 mA h g^−1^ at the current density of 100 mA g^−1^ (Figure S7, Supporting Information).

Analyses of XPS were carried out to investigate the chemical interactions between RP, MWCNT, and KB after a milling process. **Figure** **3**a shows the C1s spectrum of RP/C electrode. The C1s spectrum is fitted and separated into three peaks at 284.6, 285.3, and 286.6 eV. The first two peaks are assigned to the sp^2^ C—C and sp^3^ C—C bond, respectively, according to the previous literature,[[qv: 9b,12]] while the last peak is assigned to the C—O bond that is contributed by NaCMC (Figure S8a shows the C1s spectrum of NaCMC). This fitted result consists with the C1s spectrum of MWCNT/KB electrode (Figure S8b, Supporting Information) prepared by the same WBM process. On the other hand, a step of 12 h dry ball milling (DBM) was carried out, where an additional peak located at around 283.7 eV is differ from the result of WBM‐RP/C electrode, as shown in Figure 3b,c. According to the previous literature,^12^ we speculate that this peak might be ascribed to the P—C bond causing the shift of peak in C1s spectra of XPS. However, the possibility of this shift resulted from other bondings cannot be ruled out. Surprisingly, the DBM‐RP/C electrode only delivered a specific capacity smaller than 10 mA h g^−1^ (Figure 3d), showing the presence of P—C bond does not enhance the electrodes' performance. This result is in contrary to the beneficial effect of P—C bonds on anode performance for both LIBs and Na‐ion batteries (NIBs).[[qv: 9b,12b,13]] Moreover, Wu et al. show that BP‐graphite composites with P—C bonds have been shown an effective anode for PIBs.[[qv: 7a]] We also fabricated a DBM‐RP/graphite electrode with P—C bonds and it exhibited a specific capacity of around 200 mA h g^−1^ (Figure S9, Supporting Information). Therefore, we speculated that reversible reactions between graphite and K‐ions might break the P—C bonds to facilitate RP's alloying reaction. Even though RP/C powders were further went through a 12 h WBM process to reduce the particles size and increase the dispersion of the slurry, the electrode still showed an inert behavior to the K‐ions, resulting in a low specific capacity of <30 mA h g^−1^. Figure 3e shows the voltage profile corresponding to Figure 3d. WBM‐RP/C exhibited apparent plateaus in the region between 0.01 and 2.5 V in both discharge and charge process, corresponding to the stepwise potassiation and depotassiation process, respectively. These plateaus were disappeared in DBM‐RP/C electrode, revealing that K‐ions do not react with RP, possibly due to the presence of P—C bonds. In addition to the cycling evaluation, the electrochemical behaviors among the WBM‐, DBM‐, and DBM/WBM‐RP/C electrode were investigated by the CV test and electrochemical impedance spectroscopy (EIS) measurement (Figure 3f,g). In a CV scan, two strong peaks at 0.25 (cathodic scan) and 0.7 V (anodic scan) were obtained corresponding to RP/K‐ions reactions in WBM‐RP/C electrode, whereas extremely weak peaks were measured at the same position in DBM‐RP/C and DBM/WBM‐RP/C electrodes. Moreover, the Nyquist plots show that the charge transfer resistance of DBM‐RP/C and DBM/WBM‐RP/C electrodes was much larger than the WBM‐RP/C electrode, indicating that the K‐ions were difficult to access into RP from the electrolyte. Figure S10 in the Supporting Information presents the SEM images and XRD pattern of DBM‐RP/C powders. DBM‐RP/C powders have broad sizes ranging from 500 nm to 5 µm that is similar to the literatures of P‐based anode for NIBs^11^ and its XRD pattern is consistent with the WBM‐RP/C. DBM‐RP/C electrode assembled for a Na half‐cell delivered a specific capacity of over 1300 mA h g^−1^ (Figure S11, Supporting Information)_,_ indicating that the optimized factor of forming a high‐performance PIB anode is quite different from LIB or NIB anodes. According to these results, we consider that whether the bonding exists between RP and carbon or other elements might be a key factor to enhance the performance of PIB RP‐based anodes. For instance, SiP_2_ 14 and FeP_2_ 15 only have one phosphorus that can react with Na‐ions, indicating that the bonding between Si or Fe and P might affect the alloying reaction. In addition, C/CoP^16^ was reported as a high‐performance PIB anode, however, its phosphorus does not contribute any capacity. Therefore, the P—C bonds possibly restrict the alloying process between the K‐ions and RP, resulting in a low K‐ions storage capability of RP.

**Figure 3:**
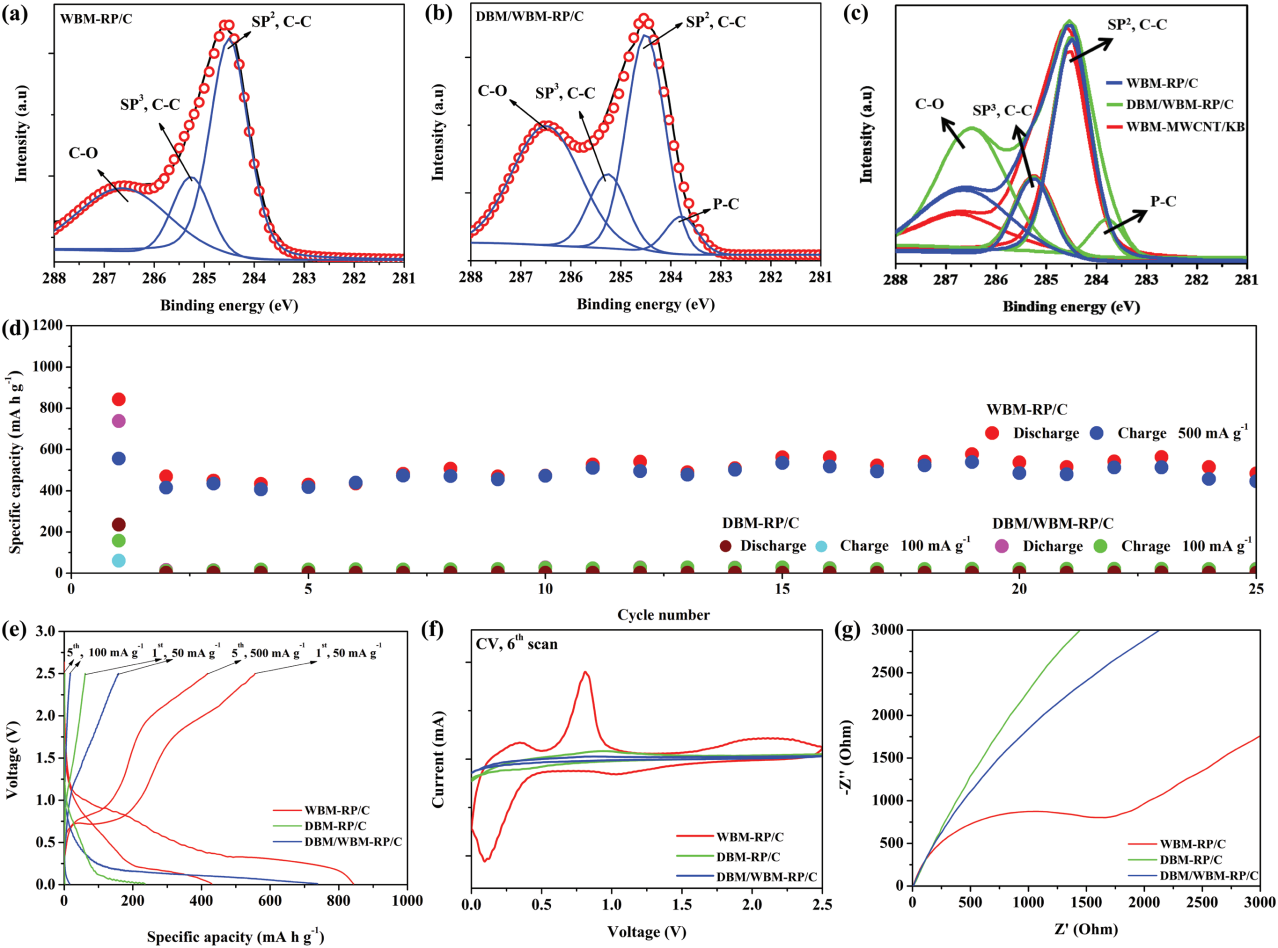
a) C1s spectrum of XPS of WBM‐RP/C. b) C1s spectrum of XPS of DBM/WBM‐RP/C. c) The comparison of C1s spectrum of XPS among WBM‐KB/CNT, WBM‐RP/C, and DBM/WBM‐RP/C. d) Cycling performance of the RP/C electrode. The red and blue dots present the cycling performance of WBM‐RP/C electrode at the current density of 500 mA g^−1^. The brown and light blue dots present the cycling performance of DBM‐RP/C electrode at the current density of 100 mA g^−1^. The purple and green dots present the cycling performance of DBM/WBM‐RP/C electrode at the current density of 100 mA g^−1^. e) Voltage profiles depict the curves of first and fifth cycle corresponding to (d). f) The comparison of CV test among the WBM‐RP/C, DBM‐RP/C, and DBM/WBM‐RP/C electrode. g) Nyquist plots of the WBM‐RP/C, DBM‐RP/C, and DBM/WBM‐RP/C electrode after five cycles.

The potassium‐ion full cells with RP‐based anodes were built by pairing it with KMnHCF cathodes to demonstrate the feasibility of RP/C as a KIB anode. The KMnHCF powders were prepared through a facile precipitation method reported by Xue et al.[[qv: 5a]] Detailed analyses of KMnHCF, including SEM images, XRD pattern, and the electrochemical evaluation of half‐cell are presented in Figure S12 (Supporting Information). The electrolyte used in the full cell was changed to 0.7 m potassium hexafluorophosphate (KPF_6_) in EC:DEC (1:1 vol%) owing the occurrence of Al corrosion, which is induced by the FSI‐based electrolyte when the voltage was raised above 4.[[qv: 5d,17]] The electrochemical performance and the corresponding voltage profile of the RP/C half‐cells using 0.7 m KPF_6_ in EC:DEC (1:1 vol%) were presented in Figure S13 (Supporting Information), showing the plateaus that are consistent with the usage of KTFSI electrolyte, however, its reversible capacity and rate capability are relatively poor. Such issue about the electrolyte used in the PIB system should be further investigated. Figure S14 in the Supporting Information shows the simulation of the charge–discharge curves of the RP/C‐KMnHCF full cell obtained by subtracting discharge–charge curves of the RP/C half‐cell from charge–discharge curves of the KMnHCF half‐cell. The charge curve of RP/C‐KMnHCF full cell should be located at the region of 2–4.4 V, which accompanied with two apparent plateaus ranging from 2.8 to 3.5 V and 3.6 to 4 V. Similarly, the discharge curve of RP/C possesses two plateaus at around 3.25 and 2 V in the region of 0–4 V. According to the results of the simulation from half‐cells, the RP/C‐KMnHCF full cell was set to operate at the cut‐off voltage between 1 and 4.2 V, because only less than 30 mA h g^−1^ capacities could be discharged at below 1 V, and besides, the charge voltage cut at 4.2 V is to avoid the occurrence of potassium plating above 4.2 V.

As shown in **Figure** **4**a, the RP/C‐KMnHCF full cell delivered a specific charge capacity of 869 mA h g^−1^ and a specific discharge capacity of 640 mA h g^−1^ at the current density of 65 mA g^−1^ based on the total mass of RP. In the tenth cycle, RP/C‐KMnHCF full cell could still display a reversible discharge capacity of 571 mA h g^−1^, indicating that the RP‐based electrode has the viability to be a high‐capacity PIB anode. Figure 4b presents the voltage profile of the RP/C‐KMnHCF full cell, corresponding to Figure 4a. The voltage profile depicts two prominent plateaus in both the charge and discharge curves that resemble the results of the simulation; nevertheless, the second plateau located at around 2 V in the discharge curve is not as obvious as the simulation result that could be attributed to the slight mismatch of the areal capacity between the anode and the cathode. Figure S15 (Supporting Information) shows the cycle performance and the voltage profile of RP/C‐KMnHCF at the different charge/discharge rate of 150 and 600 mA g^1^, displaying an average capacity of 450 and 380 mA h g^−1^ over 40 cycles, respectively. Surprisingly, when the current density raised to 1000 mA g^−1^ (Figure 4c), RP/C‐KMnHCF full cell exhibited remarkable stability and displayed a long‐term cycling life, maintaining a reversible discharge specific capacity of 203 mA h g^−1^, corresponding to a retention of 60% after 680 cycles. The superior performance of RP/C‐KMnHCF full cell operated at 1000 mA g^−1^ can be attributed to the following causes: i) the high current density gives rise to the relatively low reversible mean capacity of <300 mA h g^−1^, resulting in a small volume expansion that makes whole structure remain intact without destroying the electrical contact between RP and conductive materials. ii) As mentioned previously, the electrolyte in the half‐cell used potassium metal is unstable and became dry after a number of cycles, leading to the rapid continuous capacity fade. Compared to the potassium metal, KMnHCF as a cathode that supplied the source of potassium ions made the separator maintain the wettability of the electrolyte in long‐term cycles (Figure S17 in the Supporting Information shows the RP/C‐KMnHCF full cell, which was disassembled after 700 cycles). Notably, the voltage profile of RP/C‐KMnHCF full cell operated at the current density of 1000 mA g^−1^ differs from the current density of 65 mA g^−1^ as well as the simulation obtained from a half‐cell. As shown in Figure S18 (Supporting Information), except the first cycle, which bears the resemblance to the simulation, the plateaus of RP/C‐KMnHCF full cell operated at 1000 mA g^−1^ moved up to around 4 and 3.5 V during charge and discharge procedures, respectively. This difference could be ascribed to the discrepant capacity between RP/C and KMnHCF. According to the resulting performance of half‐cells, the high current density brings to a decline of the specific capacity of RP/C (dropping to <300 mA h g^−1^ at 1 C = 1000 mA g^−1^), whereas KMnHCF maintained the reversible specific capacity of around 95% of capacity cycled at 0.1 C under the same C rate. This result implied that only ≈30 mA h g^−1^ of the capacity from KMnHCF required to supply for RP/C, meaning that only one‐fifth of the potassiation/depotassiation occurred for KMnHCF during the cycling process cycled at 1 C, corresponding to the charge/discharge voltage plateau in the region of 2.5–4 and 4–4.4 V, respectively. Therefore, we resimulated the charge/discharge curves of the full cell by subtracting RP/C discharge and charge curve (0.01–2.5 V) from KMnHCF charge curve of 2.5–4 V and discharge curve of 4–4.4 V, respectively, from their respective results of the half‐cells cycled at 1 C, as shown in Figure S19 (Supporting Information). The results of resimulation are consistent approximately with the voltage profiles of the full cell cycled at 1 C, showing that the charge plateau and discharge plateau raised up to around 4 and 3.5 V, respectively. As a proof of concept, we built a pouch‐type battery with RP/C anode by coupling it with KMnHCF cathode (Figure 4d). For a typical assembly, both anode and cathode were prepotassiated for three cycles in an aluminum bag using handmade potassium foil as the counter electrode. Then, the electrodes and the separator were wound together to form a 4 cm × 7.5 cm cell core of the pouch‐type battery, exhibiting a capacity of 10 mA h. The RP‐based potassium‐ion pouch‐type battery could light up more than 40 light emitting diodes (LEDs) with green and blue color that need high voltage of >3 V to be drove up, as shown in Figure 4e.

**Figure 4:**
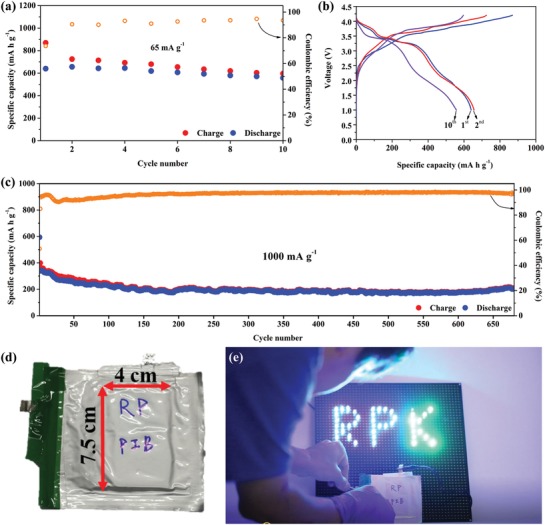
Electrochemical performance and the practical application of RP/C‐KMnHCF full cells. a) Cycling performance of RP/C‐KMnHCF coin full cell at the current density of 65 mA g^−1^. b) Voltage profile of RP/C‐KMnHCF coin full cell at the current density of 65 mA g^−1^ corresponding to (a). c) Cycling performance of RP/C‐KMnHCF coin full cell at the current density of 1000 mA g^−1^ over 680 cycles. The specific capacity and the current density were calculated based on the mass of RP. The corresponding specific capacity calculated based on the total mass of active materials of anode and cathode was shown in Figure S16 in the Supporting Information. d) A 10 mA h pouch‐type battery, which contained a 4 × 7.5 cm cell core built by the configuration of RP/C‐KMnHCF. e) Pouch‐type battery lighted up over 40 pieces blue and green 3 V LED bulbs.

**Figure** **5**a,b shows the comparative plots including the data of electrochemical performance of the RP/C and other anode materials evaluated in the nonaqueous potassium‐ion system. Figure 5a presents the plots of the specific capacity against the current density based on the half‐cell data. This comparative graph reveals that RP electrode fabricated by a facile one‐pot WBM possesses a better specific capacity and a high‐rate capability as compared to most of the P‐ or C‐based electrodes. Furthermore, Figure 5b depicts a comparison of the potassium‐ion full cells, showing the superiority of the configuration of RP/C as anode and KMnHCF as cathode. Consequently, the RP/C‐KMnHCF full cell can output a specific energy density of 193 Wh kg^−1^ that is higher than the previous reported PIBs (Figure S21a and Table S3, Supporting Information). Notably, without the prepotassiation process, the energy density of RP/C‐KMnHCF full cell might decrease to 157 Wh kg^−1^ (Figure S21b, Supporting Information) because the cathode requires an extra mass loading to satisfy the large irreversible capacity of anode in the initial cycle (the value of simulated energy density is calculated based on the Columbic efficiency of 68% in the first cycle). This configuration demonstrates a promising candidate that enables the PIB to have the high working voltage, high power, sufficient energy density, and long‐term cycling life to be an available alternative option for the rechargeable battery system.

**Figure 5:**
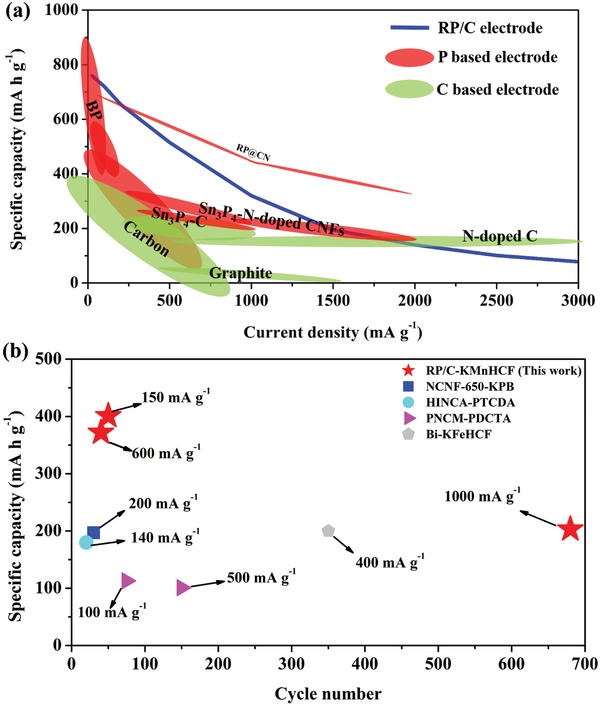
Comparative plots of the RP/C and other anode materials evaluated in nonaqueous potassium‐ion systems. a) Rate performance comparison of the RP/C electrode and previous anode materials[[qv: 3b,6a,c-e,7,16,18]] for PIBs. The plots depicted the charge specific capacity against the current density based on the half‐cell data. (The specific capacity was obtained via calculating the mass of the active materials.) Detailed plots and data were shown in Figure S20 and Table S1 in the Supporting Information. b) Cycling performance comparison of the RP/C‐KMnHCF full cell and other previous configuration of the potassium‐ion full cells.[[qv: 6d,e,19]] The specific capacity and the current density were calculated based on the mass of active materials of anode. Detailed data were shown in Table S2 in the Supporting Information.

In summary, RP was successfully activated for PIB anodes via a facile one‐pot WBM process. Nanoscale RP particles supported by the conductive network composed of MWCNT and KB delivered a high reversible capacity and good rate capability, enabling the RP to be an appealing K‐ions storage anode material. From the analysis of XPS spectrum and electrochemical measurements, it is speculative to suggest that no P—C bonds formation can reduce the resistance of the alloying reaction between RP and K‐ions and obtain activated RP‐based anode materials for PIBs. A full cell comprising an RP/C anode and a KMnHCF cathode gives a long‐term cycling life (680 cycles), a high average working voltage (≈3.4 V), and a specific energy density (193 Wh kg^−1^) surpassing all of the reported K‐ions full cells based on the total mass of active materials of anode and cathode, revealing that this configuration is a promising option for next‐generation sustainable battery systems.

## Conflict of Interest

The authors declare no conflict of interest.

## Supporting information

Supplementary
