# Supplementary material for: Red Phosphorus Potassium‐Ion Battery Anodes
Source: Adv Sci (Weinh). 2019 Feb 28;6(9):1801354. doi: 10.1002/advs.201801354 (PMC6498114; doi:10.1002/advs.201801354)
Supplement: Supplementary file 1 — Supplementary [file ADVS-6-1801354-s001.pdf]

Copyright WILEY-VCH Verlag GmbH & Co. KGaA, 69469 Weinheim, Germany, 2019.

ADVANCED SCIENCE
Open Access

# Supporting Information

for *Adv. Sci.,* DOI: 10.1002/advs.201801354

## Red Phosphorus Potassium-Ion Battery Anodes

*Wei-Chung Chang, Jen-Hsuan Wu, Kuan-Ting Chen, and Hsing-Yu Tuan**

Copyright WILEY-VCH Verlag GmbH & Co. KGaA, 69469 Weinheim, Germany, 2018.

# Supporting Information

**Red Phosphorus Potassium-Ion Battery Anodes**

Wei-Chung Chang, Jen-Hsuan Wu, Kuan-Ting Chen and Hsing-Yu Tuan*

Department of Chemical Engineering, National Tsing Hua University, 101, Section 2, Kuang-Fu Road, Hsinchu, Taiwan 30013, ROC

*Corresponding authors

Phone: (886)3-571-5131 ext:42509

Email: hytuan@che.nthu.edu.tw

**Experimental section**

**Materials**

Ethanol (99.8%), Sodium carboxymethyl cellulose (NaCMC, average Mw ~700000), poly (acrylic acid) (PAA, average Mv~450000), potassium metal (chunks in mineral oil, 98%), potassium hexacyanoferrate(II) trihydrate ($K_4Fe(CN)_6 \cdot 3H_2O$, 98.5%), potassium chloride (KCl, 99%), manganese(II) nitrate solution ($Mn(NO_3)_2$ 45~50 wt% in dilute nitric acid), potassium hexafluorophosphate ($KPF_6$, 99.5%), diethyl carbonate (DEC, 99%) were purchased from Sigma-Aldrich. Red phosphorus (RP, 98.9%) was purchased from Alfa Aesar. Multi-wall carbon nanotubes (MWCNT) was purchased form golden innovation business co. ltd. Ketjen black EC600jd was purchased from lion specialty chemicals. Potassium bis(trifluoromethanesulfonyl) imide (KTFSI, 99.8%) was purchased from kanto chemical. Copper metal foil (10 um), ethylene carbonate (EC), fluoroethylene carbonate (FEC), coin-type cell CR2032 were purchased from shining energy. Glass fibers (diameter = 19 mm) were purchased from Advantec. The components of pouch type battery were purchased from MTI Shenzhen kejingtar technology.

**Fabrication of RP/C electrode**

In a typical fabrication, RP, MWCNT, KB, NaCMC were added into a stainless steel in the ratio of 64: 8: 8: 20. Then, DI water was injected into the stainless steel to form a slurry with 10% solid content. After that, the stain steel jar was put into the Planetary Ball Mill PM 100 machine and rotated at 300 rpm for 12 hours. A deep red color homogenous slurry could be obtained after 12 hours wet ball milling process. The slurry was then coated onto the copper foil to from a RP/C electrode with the areal RP mass loading of 0.75-1.1 mg $cm^{-2}$. The RP/C electrode was then dried at 100 °C under argon gas to remove residual water. Afterward, the RP/C electrode was pressed by a rolling machine, followed by tailoring into suitable size for half-cell assembly.

**Synthesis of KMnHCF**

The synthesis of KMnHCF was used a typical precipitation method reported by Xue et al.[1] First, 2.3 g $Mn(NO_3)_2$ solution (50 wt.%) dissolved into 50 mL DI water and 1.27 g $K_4Fe(CN)_6 \cdot 3H_2O$, 20 g KCl dissolved into 100 mL DI water were prepared. Next, these two solutions were slowly dropped together under magnetic stirring at 50 °C for 2 hours. Then, the product was centrifugalized and washed with DI water several times to remove the residual by-products.

**Preparation of KMnHCF electrode**

300 mg active materials K-MnHCFe (62.5 %), 90 mg super P (18.75 %), 90 mg PAA (18.75 %) were mixed with ethanol to form a homogeneous slurry by using planetary mixer for 3 hours. The slurry was then coated onto an aluminum foil, and dried at 100 °C under argon gas. The areal mass loading of KMnHCF electrode is 4.5~5.5 mg $cm^{-2}$ (active material).

**Characterization**

The investigations of materials were carried out by using scanning scanning electron microscopy (HITACHI-SU8010) with energy-dispersive X-ray spectroscopy (HORIBA, EX-250), X-ray diffraction (Rigaku Ultima IV), Raman spectroscopy (LABRAM HR 800 UV) with a radiation of 514 nm. X-ray photoelectron spectra (XPS) were obtained by using PHI Quantera SXM. All of the spectra obtained from XPS analysis were first calibrated by referencing binding energy of C1s (284.6 eV), followed by the curve fitting using the software of XPSPEAK VER. 4.1.

**Electrochemical characterization**

**RP/C electrode**

The electrochemical performance of the RP/C electrodes were evaluated in the CR2032 coin-cells with the handmade potassium metal foil as the counter electrode. The weight of electrode was measured using a microbalance with 0.1 μg resolution (Sartorius SE2) to obtain an accurate weight of active material. The specific capacity is calculated based on the mass

loading of active materials (RP). The CR2032 half-cells were assembled in an argon-filled glove box using RP/C as the working electrode, handmade potassium metal foil and glass fiber as the separator. The main electrolyte added in the RP/C coin half-cells was 1 M KTFSI in ethylene carbonate (EC)/diethyl carbonate (DEC) (EC/DEC) (1:1 vol%). The electrochemical performance of RP/C electrodes using 0.7 M $KPF_6$ in ethylene carbonate (EC)/diethyl carbonate (DEC) (EC/DEC) (1:1 vol%) as the electrolyte was also evaluated. The data of electrochemical performance of RP/C electrode were recorded using Maccor Series 4000 battery test system within the voltage of 0.01-2.5 V (vs. $K/K^+$).

**KMnHCF electrode**

The electrochemical performance of KMnHCF coin half-cells was evaluated using handmade potassium metal foil as the counter electrode and 0.7 M $KPF_6$ in ethylene carbonate (EC)/diethyl carbonate (DEC) (EC/DEC) (1:1 vol%) as the electrolyte. The data of electrochemical performance of KMnHCF electrode were recorded using Maccor Series 4000 battery test system within the voltage of 2-4.4 V (vs. $K/K^+$).

**Coin full cell and pouch type battery**

The CR2032 coin full cell was assembled by using RP/C as an anode and KMnHCF as a cathode with an A/C ratio of 1.05~1.1 based on the real capacity. Before the assembly, to pair the anode and cathode precisely, both RP/C and KMnHCF electrode were pre-potassiation for 3 cycles. The pre-potassiation procedures of RP/C and KMnHCF electrode are as same as the setting of their measurements in half-cells. The pre-potassiation electrodes were gone through 3 complete charge-discharge cycles at the rate of 0.1 C before taking out from the half-cells. (The cut-off voltage of RP/C and KMnHCF is 2.5 V and 2 V, respectively.) Then, the RP/C anode was selected with a real capacity that was 5~10 % higher than KMnHCF cathode for assembly, avoiding the occurrence of potassium plating on the surface of anode side. However, the extra loading mass of RP/C anode, which might not storage K-ions, result a slight decrease in energy density. The electrolyte added in the full cell was 0.7 M $KPF_6$ in

ethylene carbonate (EC)/diethyl carbonate (DEC) (EC/DEC) (1:1 vol%). For a typical assembly of pouch type battery, a RP/C anode and KMnHCF cathode tailored into the area of 45 $cm^2$ (3 cm * 15 cm) were prepared. Then, the aluminum tab and the nickel tab were connected to the cathode and the anode, respectively. Both anode and cathode were pre-potassiation for 3 cycles in an aluminum bag using handmade potassium foil as the counter electrode. After that, the anode, the cathode and the separator were wound together in the argon-filled glove box and put into an aluminum bag, followed by the injection of electrolyte and the vacuum sealing. The data of electrochemical performance of RP/C-KMnHCF full cells were recorded using Maccor Series 4000 battery test system under the cut-off voltage window between 1 and 4.2 V.

**Calculation of theoretical capacity of KP alloy**

$K^+ + e^- + P \rightarrow KP$

1 mole electrons = 96500 C (coulombs), 1 C = 0.2777 mA h

Atomic weight of phosphorus = 30.9737

Theoretical specific capacity of KP $= \frac{1\times96500\times0.2777}{30.9737} = 865.187$ mA h $g^{-1}$

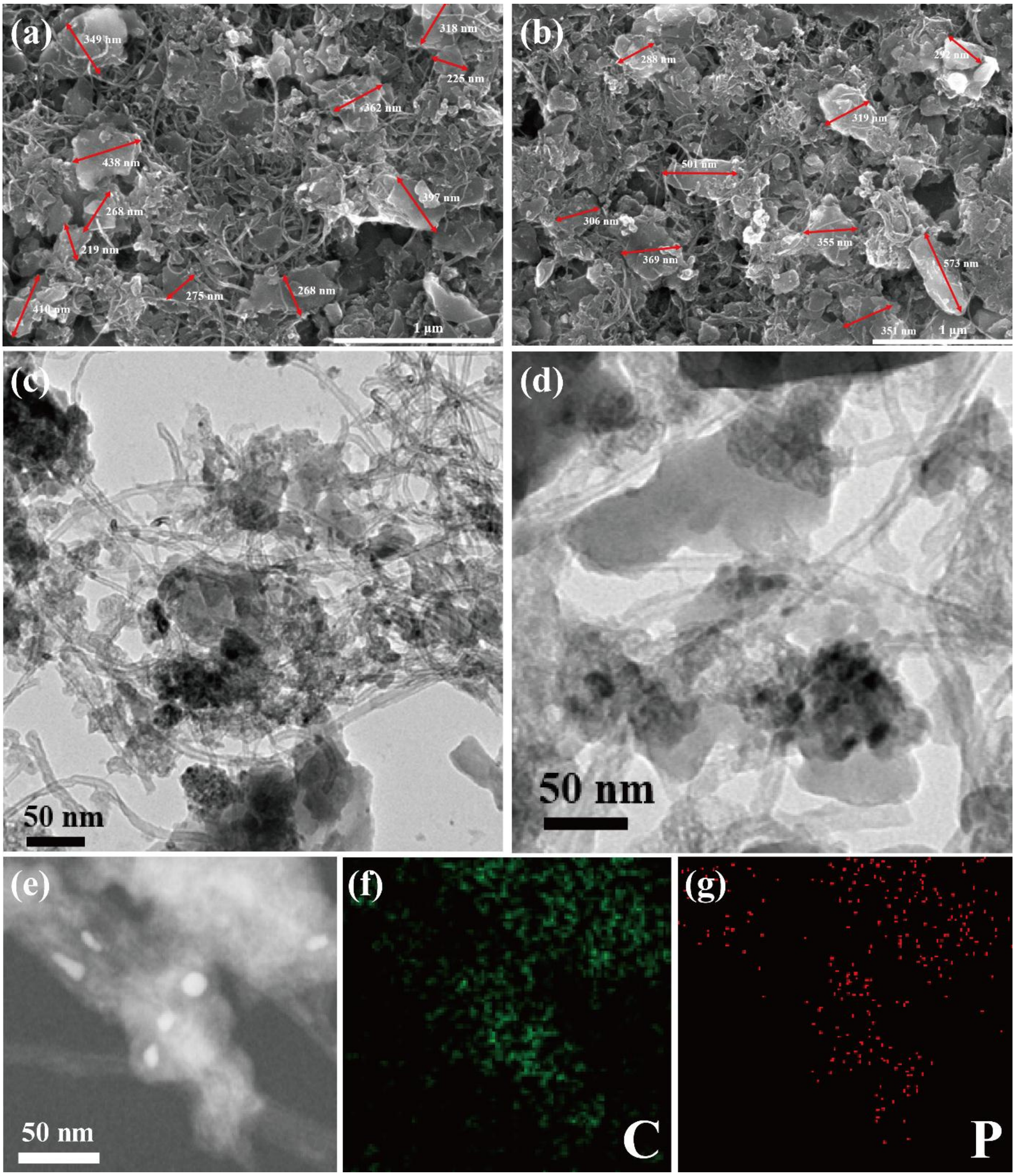


**Figure S1.** (a), (b) Size measurements of WBM-RP/C. (c), (d) TEM images of WBM-RP/C. (e) High magnification TEM image of WBM-RP/C. (f), (g) EDS mapping images corresponding to (e). The green and red dots represent carbon and phosphorus, respectively.

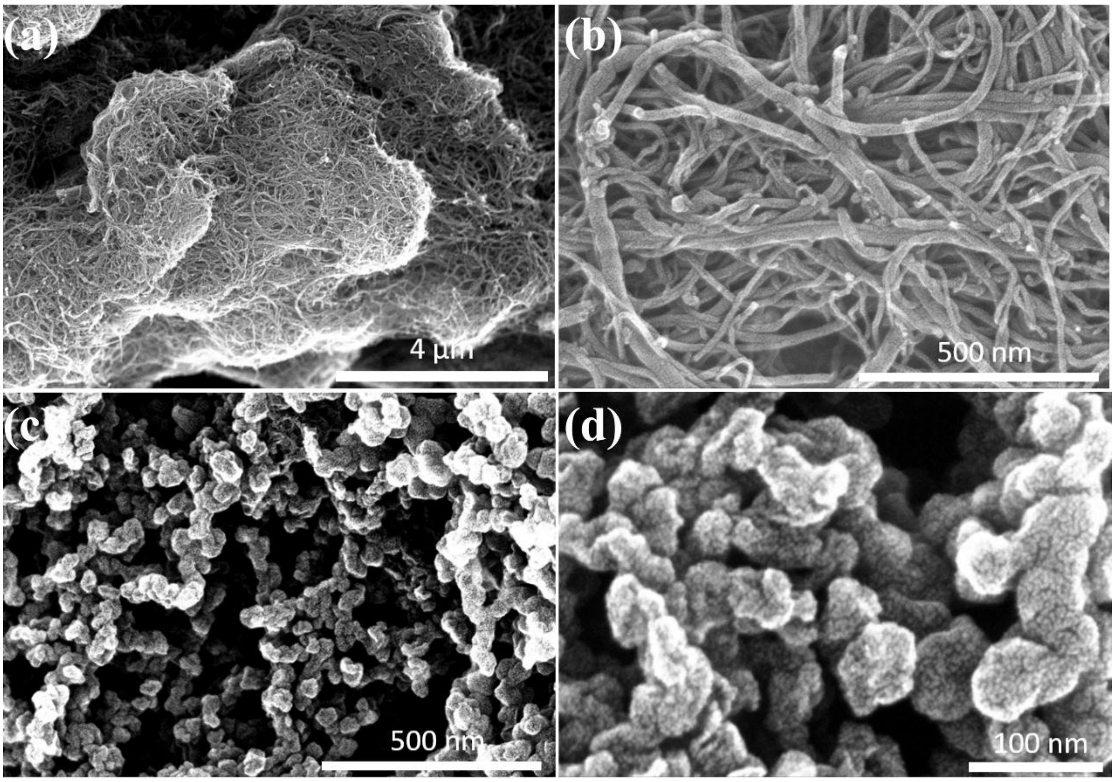


**Figure S2.** (a-b) SEM images of MWNCTs. (c-d) SEM images of Ketjen black.

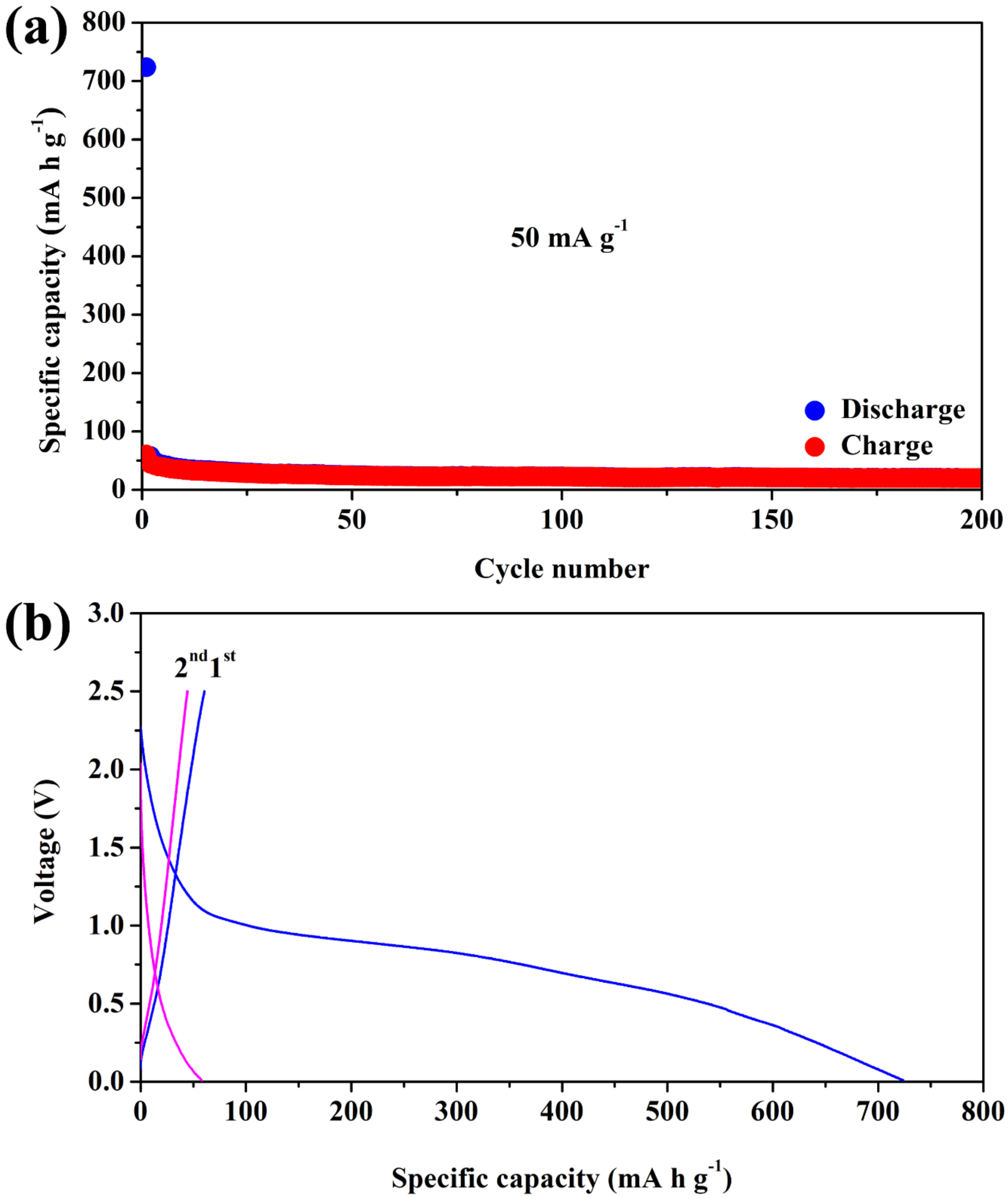


**Figure S3.** (a) Electrochemical performance of MWCNT/KB half-cell. (b) Voltage profile corresponding to (a)

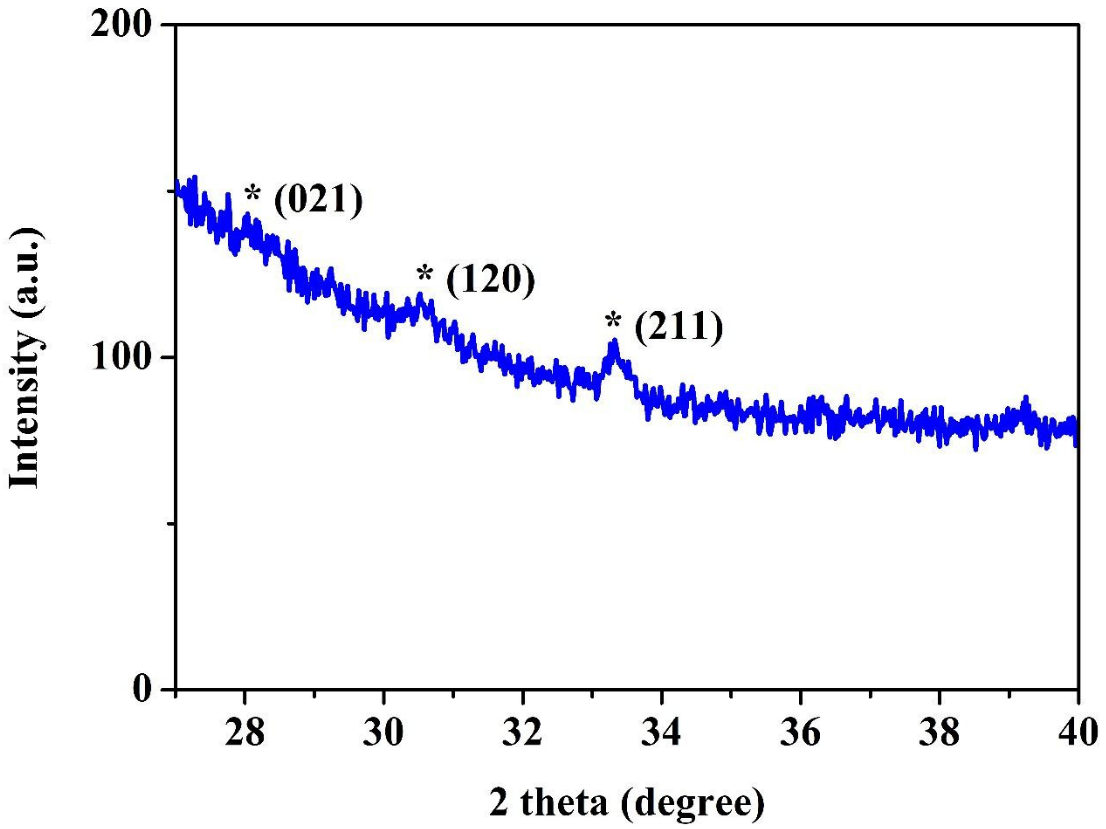


**Figure S4.** Ex-situ XRD pattern of RP/C electrode after first discharge process at the current density of 25 mA $g^{-1}$. (JCPDS 04-004-2804)

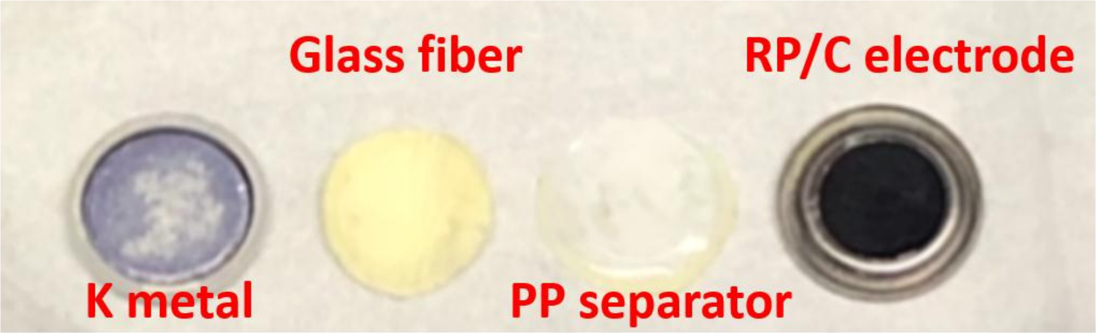


**Figure S5.** The image of a disassembled coin half-cell corresponding to Figure 2f.

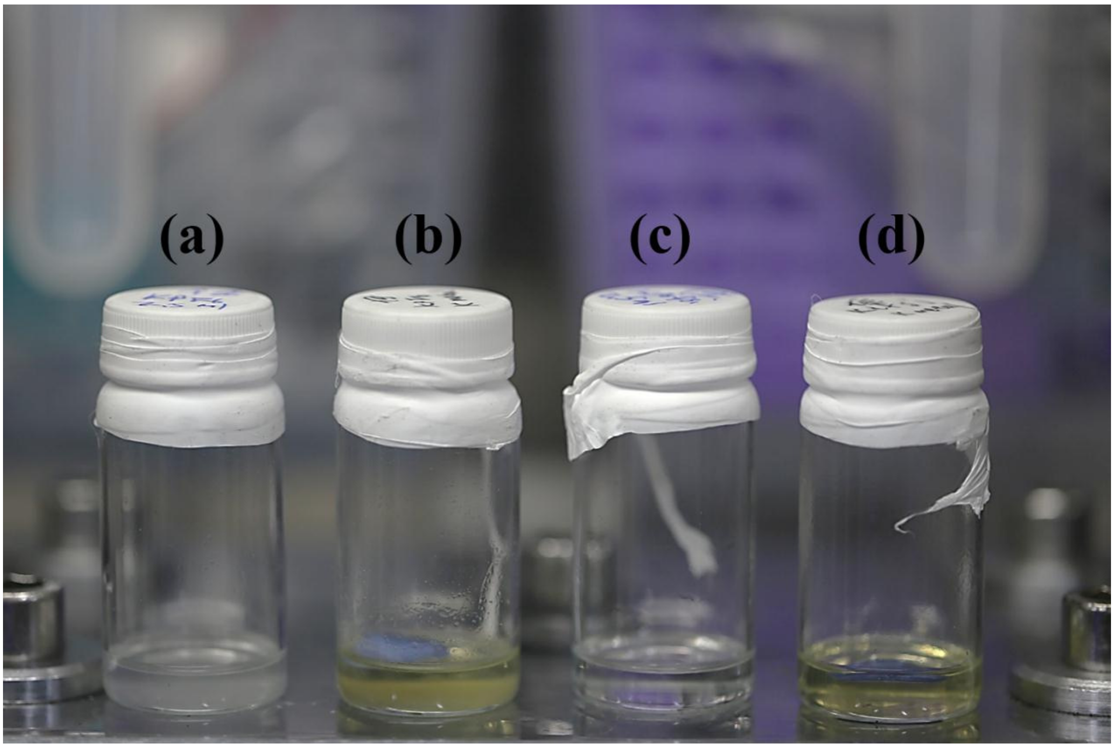


**Figure S6.** The image of electrolyte soaked with/without potassium metal after 5 days. (a) 0.7 M $KPF_6$ in EC/DEC (1:1 vol%) without potassium metal. (b) 0.7 M $KPF_6$ in EC/DEC (1:1 vol%) with potassium metal. (c) 1M KTFSI in EC/DEC (1:1 vol%) without potassium metal. (d) 1M KTFSI in EC/DEC (1:1 vol%) soaked with potassium metal.

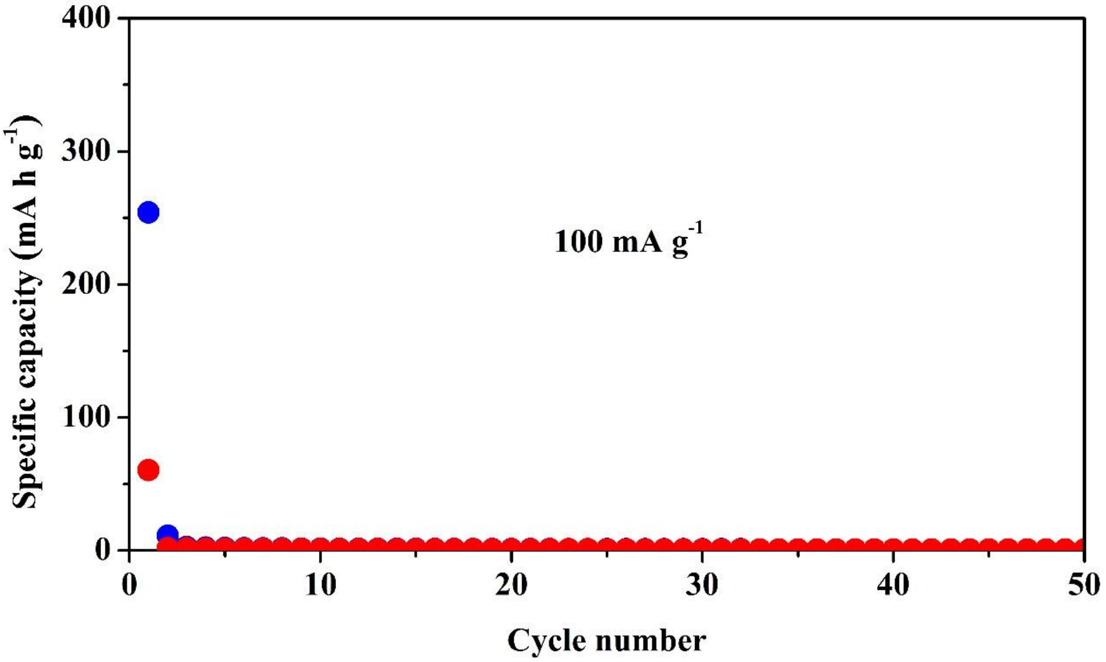


**Figure S7.** The electrochemical performance of RP/C using the electrolyte of 1M KTFSI in EC/DEC (1:1 vol%) + 5% FEC.

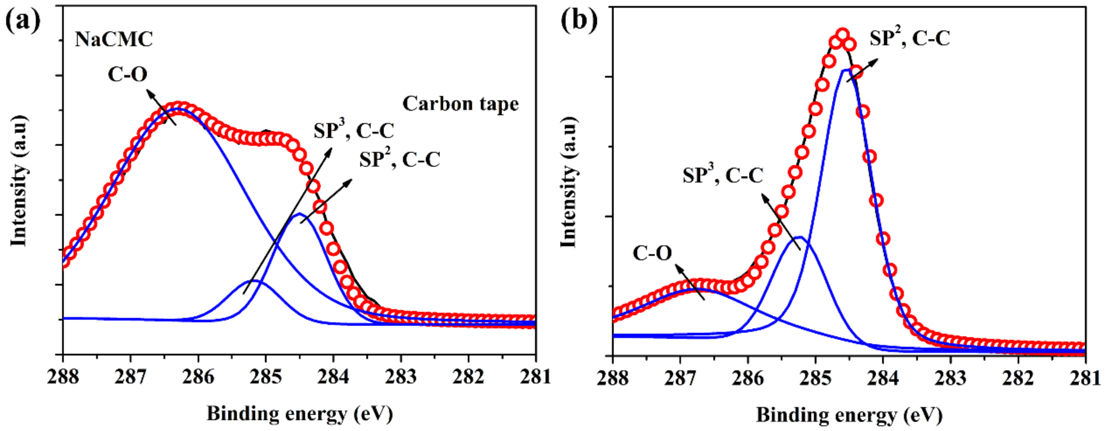


**Figure S8.** (a) C1s spectrum of XPS of NaCMC powder. (b) C1s spectrum of XPS of MWCNT/KB electrode.

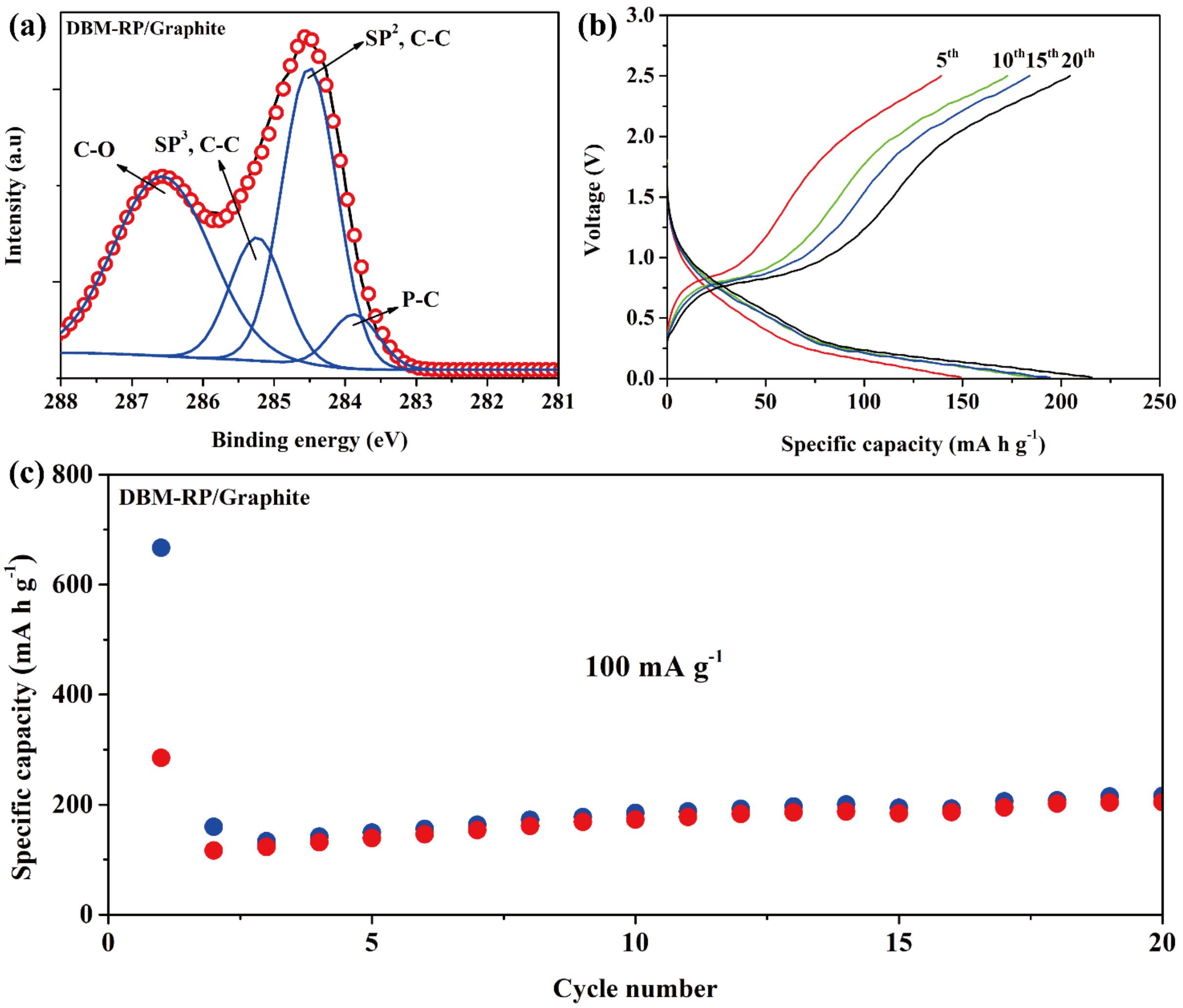


**Figure S9.** Characterization of DBM-RP/Graphite. (a) C1s spectrum of XPS (b) Voltage profile corresponding to (c). (c) Cycling performance of DBM-RP/Graphite K half-cell.

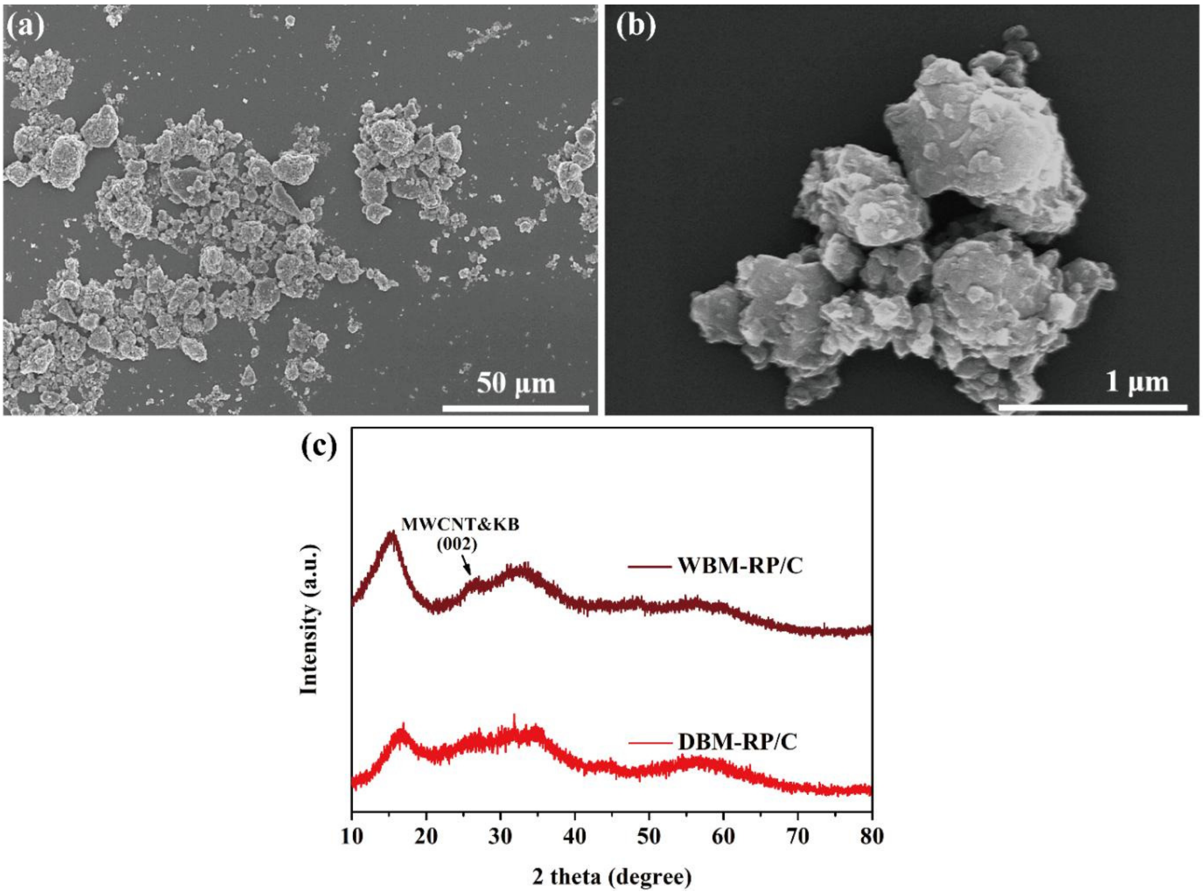


**Figure S10.** Structural analyses of DBM-RP/C. (a) Low magnification SEM image. (b) High magnification SEM image. (c) XRD pattern.

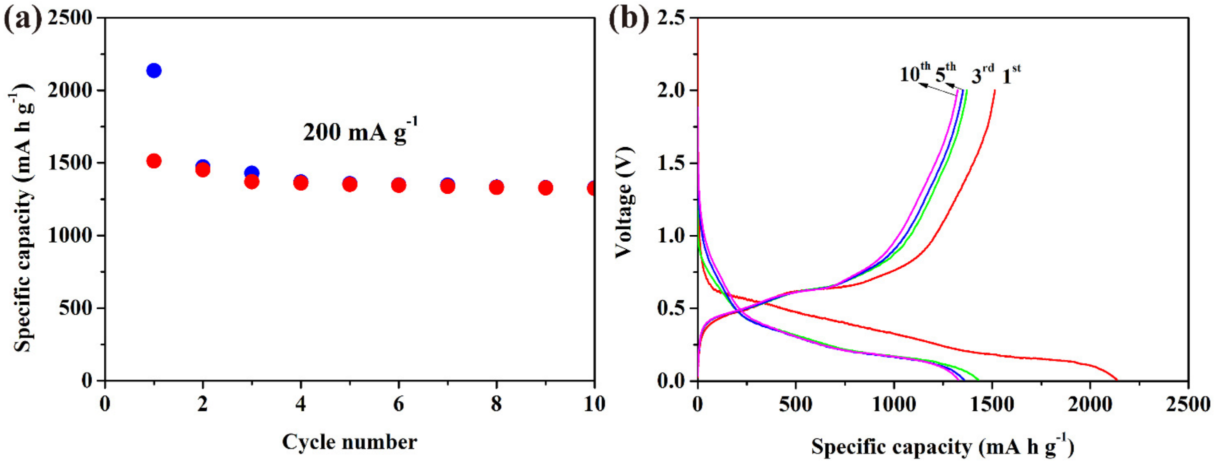


**Figure S11.** Electrochemical performance of DBM-RP/C Na half-cell. (a) Cycling performance. (b) Voltage profile. DBM-RP/C Na half-cell was evaluated within the voltage of 0.01-2V using the electrolyte of 1M $NaPF_6$ in EC: DEC: FEC = 5:5:1 (vol%).

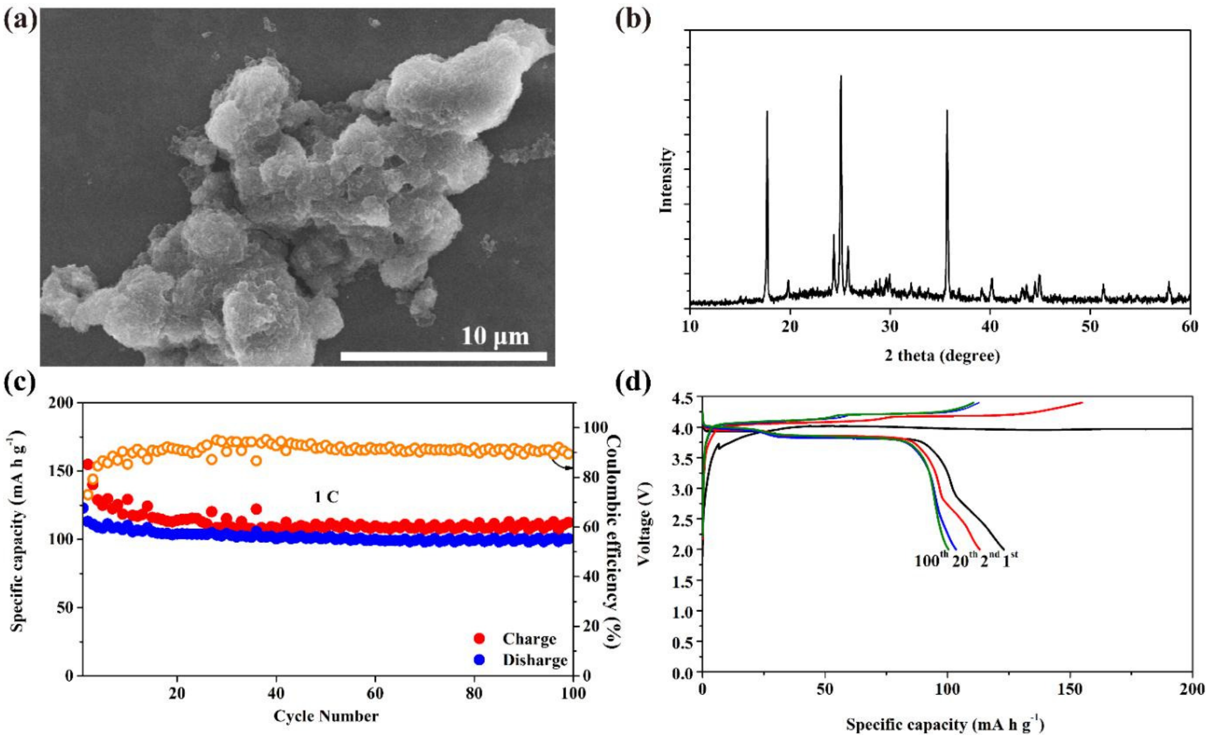


**Figure S12.** Analysis of KMnHCF. (a) SEM image of KMnHCF powder. (b) XRD pattern of KMnHCF powder. The result is consistent with the XRD pattern reported by Xue et al. (c) Cycling performance of KMnHCF half-cell at the rate of 1 C over 100 cycles. 1 C = 150 mA $g^{-1}$. (d) Voltage profile of KMnHCF half-cell corresponding (c).

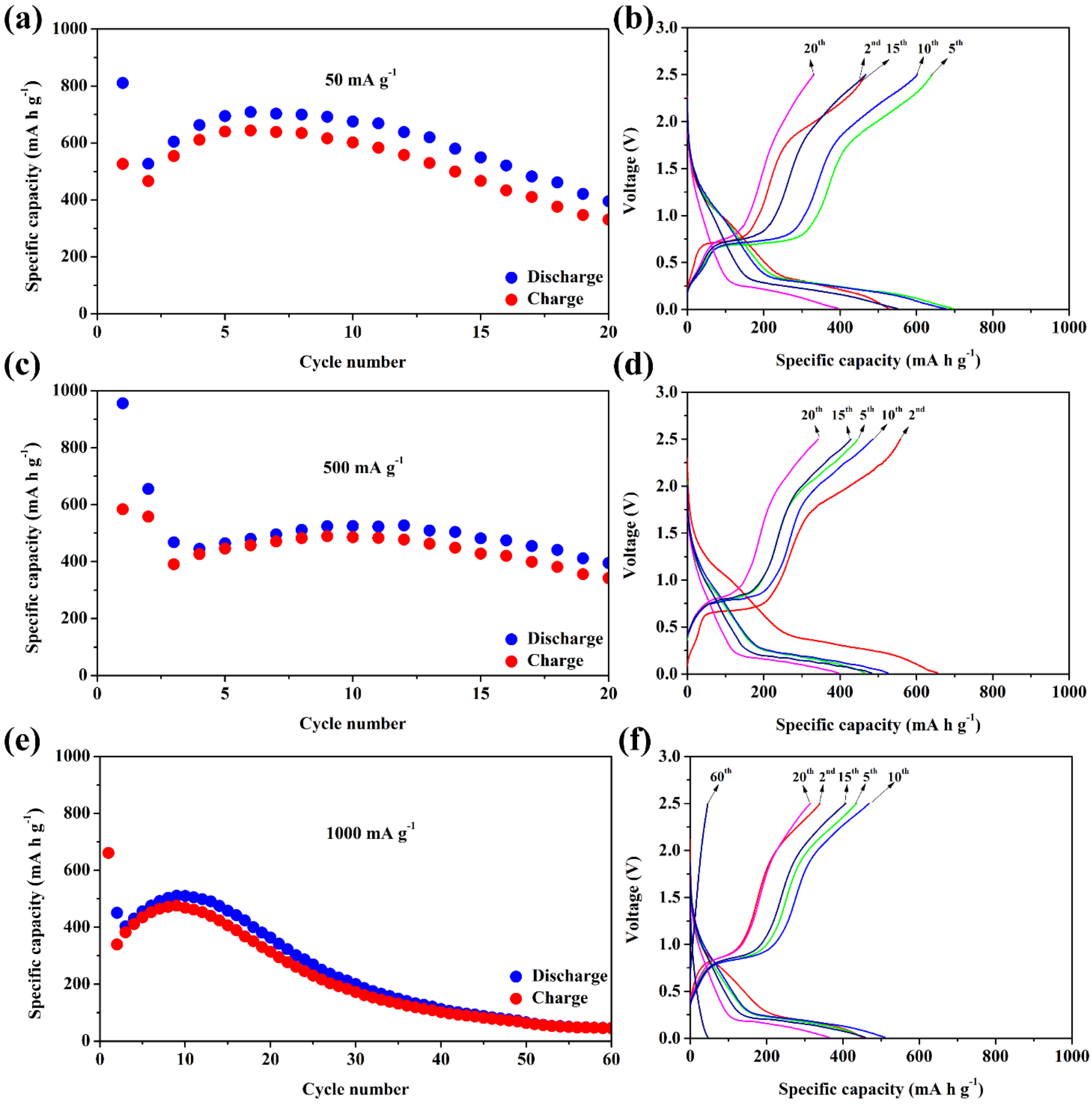


**Figure S13.** Electrochemical performance of RP/C electrodes using 0.7 M $KPF_6$ in EC/DEC (1:1 vol%). The RP/C electrodes were evaluated at various current density of (a) 50 mA $g^{-1}$. (c) 500 mA $g^{-1}$. (e) 1000 mA $g^{-1}$. (b), (d) and (f) are the voltage profiles corresponding to (a), (c) and (e), respectively.

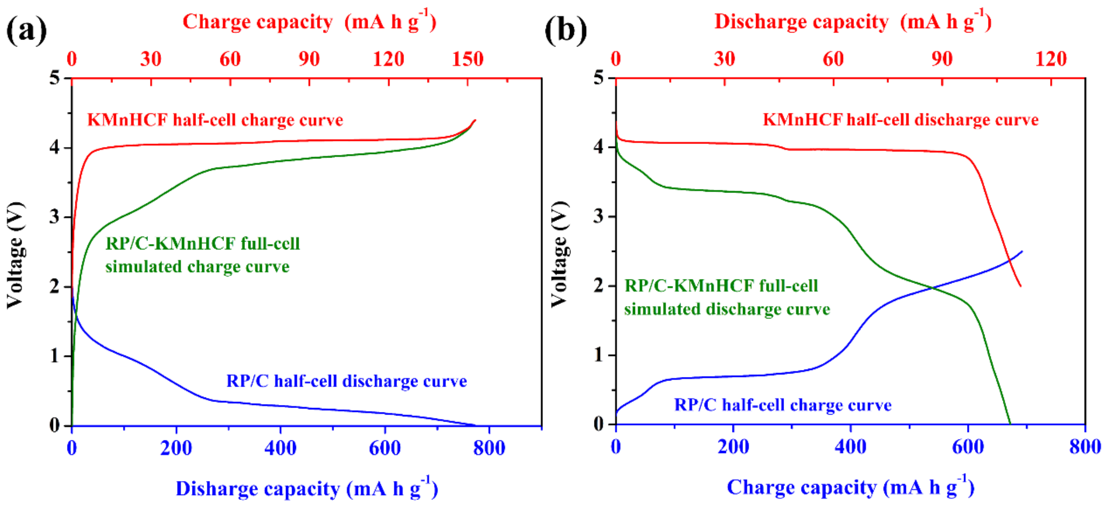


**Figure S14.** Simulation of charge-discharge curves of the RP/C-KMnHCF full-cell. (a) Simulation of charge curve of RP/C-KMnHCF full-cell depicted by subtracting RP/C half-cell discharge curve from KMnHCF half-cell charge curve. (b) Simulation of discharge curve of RP/C-KMnHCF full-cell depicted by subtracting RP/C half-cell charge curve from KMnHCF half-cell discharge curve.

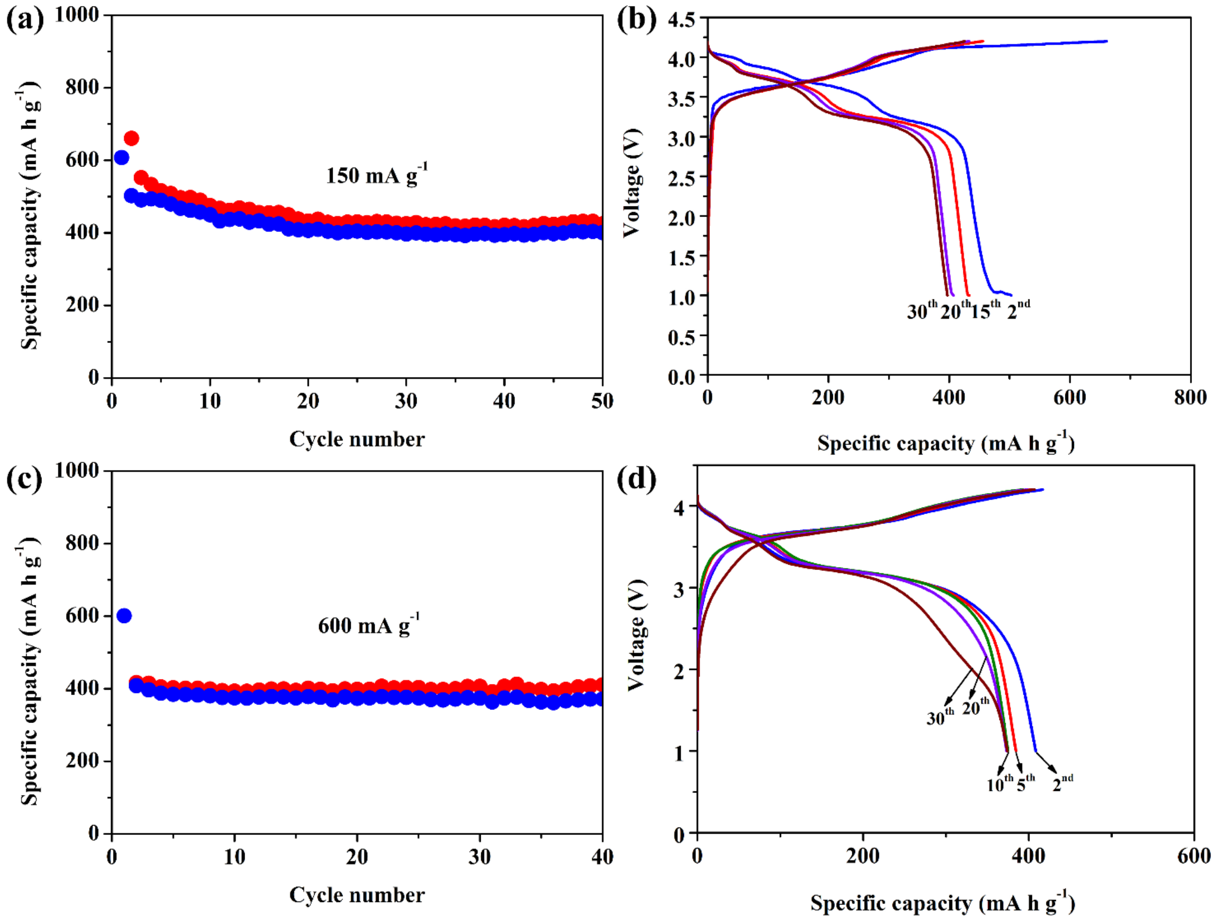


**Figure S15.** Electrochemical performance of RP/C-KMnHCF full cell at the various charge/discharge current density. (a) RP/C-KMnHCF full cell cycled at 150 mA g$^{-1}$ over 50 cycles. (b) Voltage profile of RP/C-KMnHCF full cell at 150 mA g$^{-1}$ corresponding to (a). (c) RP/C-KMnHCF full cell cycled at 600 mA g$^{-1}$ over 40 cycles. (d) Voltage profile of RP/C-KMnHCF full cell at 600 mA g$^{-1}$ corresponding to (c)

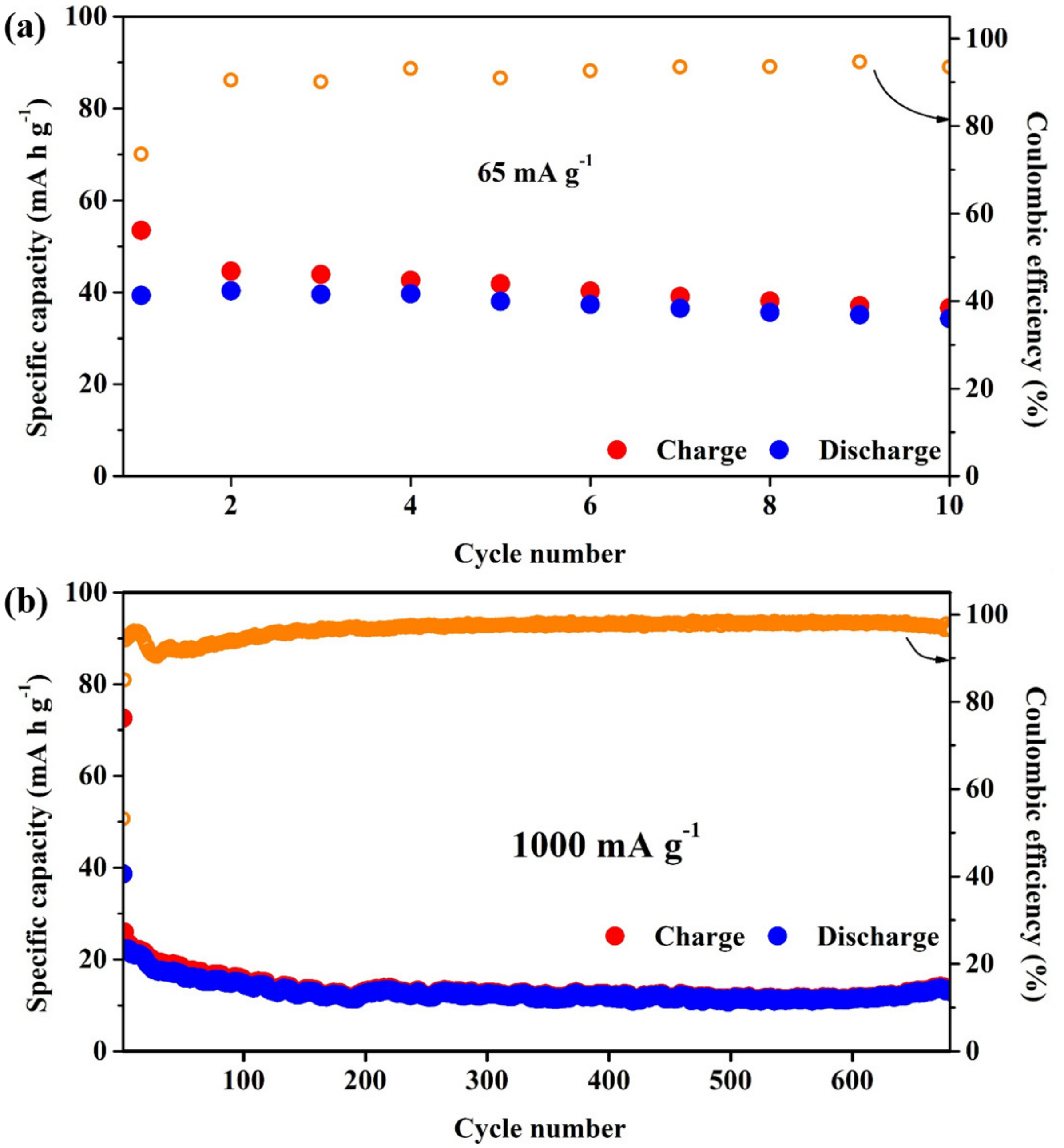


**Figure S16.** Specific capacity of RP/C-KMnHCF full cells calculated based on the total mass of active materials of anode and cathode. (a) and (b) corresponds to Figure 4a and 4c, respectively.

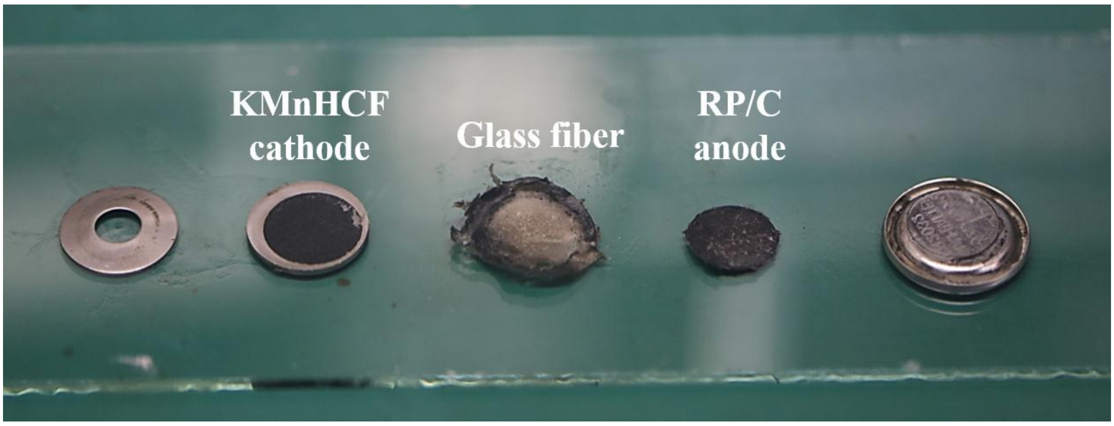


**Figure S17.** Image of disassembled RP/C-KMnHCF coin full cell after 700 cycles.

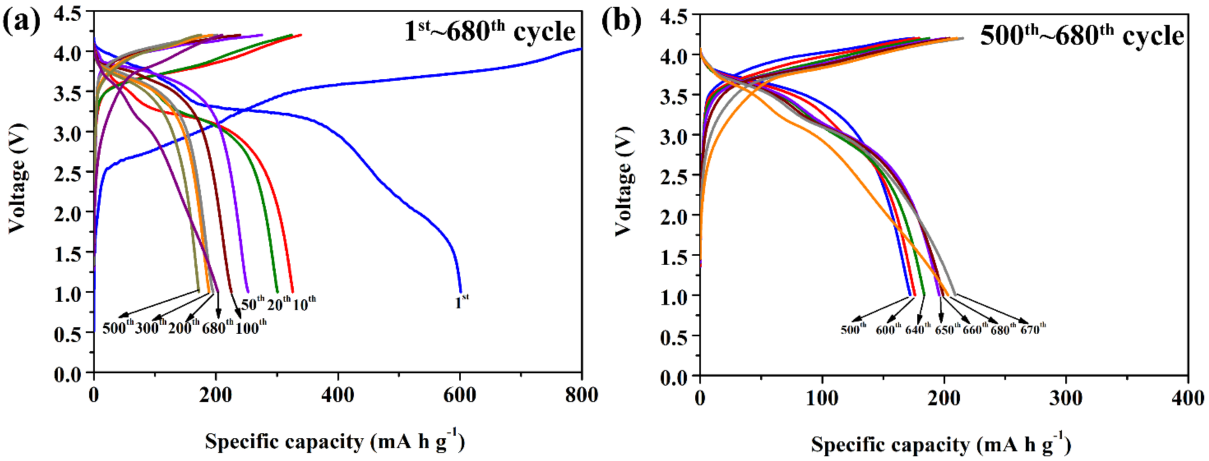


**Figure S18.** Voltage profile corresponding to Figure 4c. (a) 1$^{st}$-680$^{th}$ cycle. (b) 500$^{th}$-680$^{th}$ cycle.

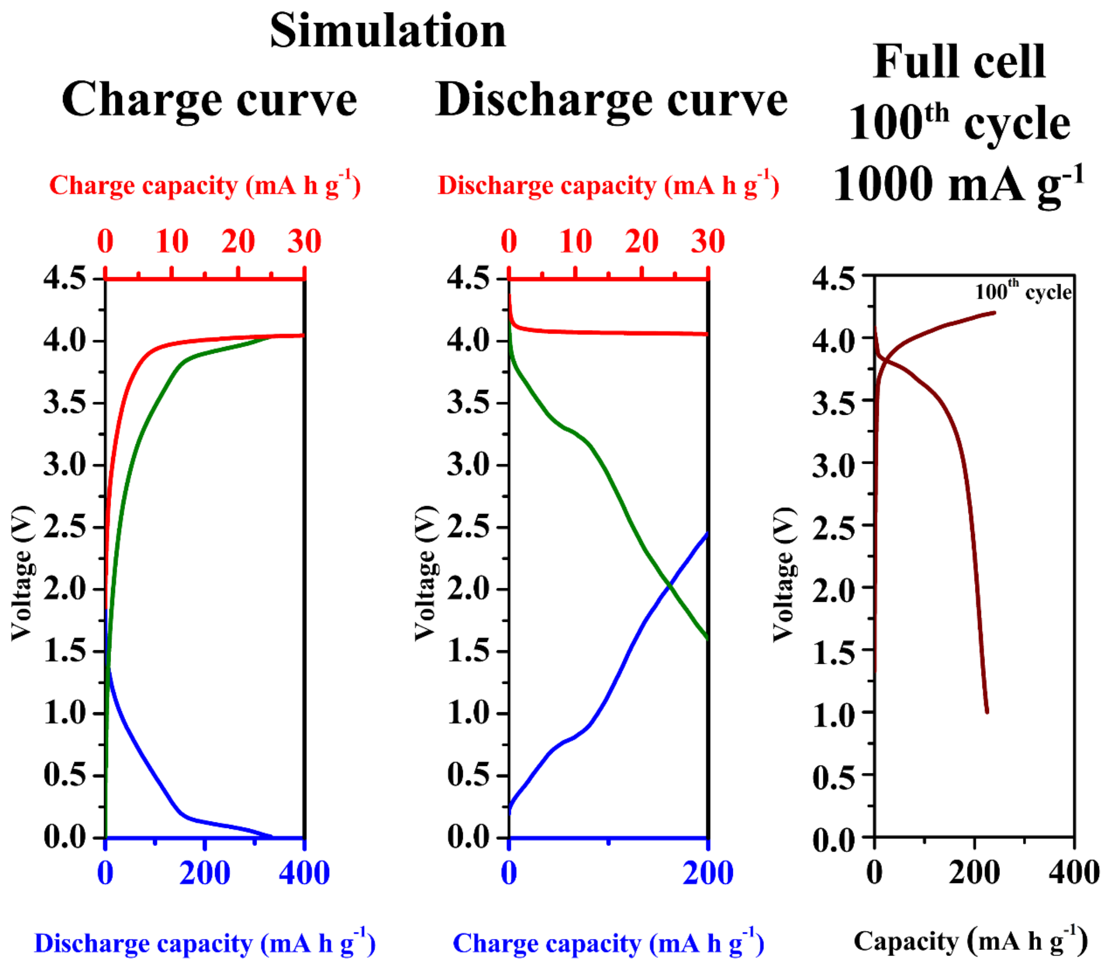


**Figure S19.** Re-simulation corresponding to Figure 4c. The simulated charge curve was re-produced by subtracting RP/C discharge curve of 0.01-2.5 V from KMHCF charge curve of 2.5-4V. The simulated discharge curve was re-produced by subtracting RP/C charge curve of 0.01-2.5 V from discharge curve of 4-4.4 V.

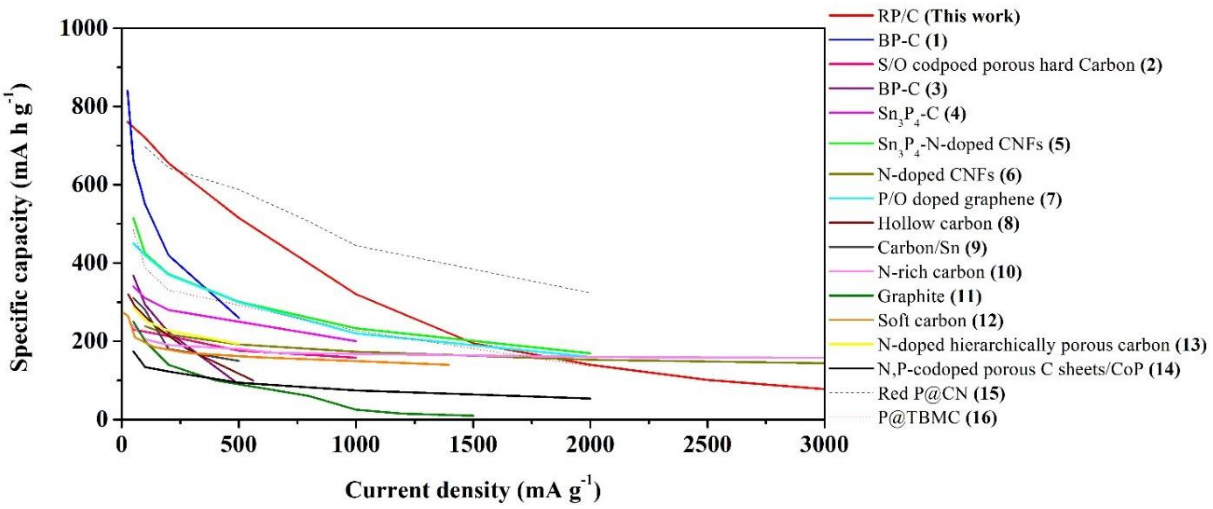


**Figure S20.** Detailed plots corresponding to Figure 5a.

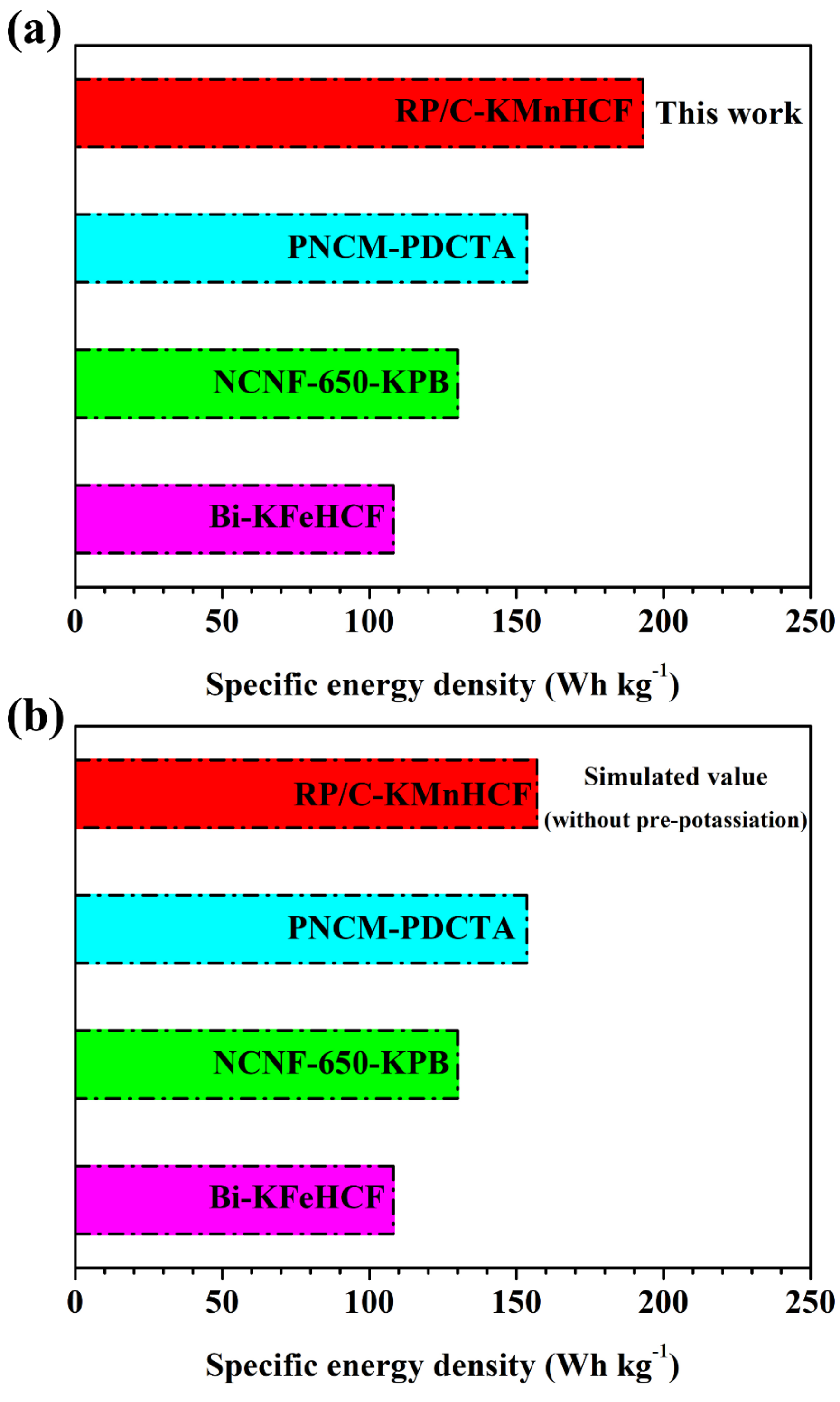


**Figure S21.** The comparison of specific energy density. (a) RP/C-KMnHCF and previous reported full-cell configurations. The specific energy density was calculated based on the total mass of active materials of cathode and anode. (b) RP/C-KMnHCF without pre-potassiation and previous reported full-cell configurations. The simulated value was calculated based on the Columbic efficiency of 68% in the first cycle.

**Table S1.** Detailed data corresponding to Figure 5a and Figure S20.

| | **Anode Material** | **Current density (mA g$^{-1}$)** | **Specific charge capacity (mA h g$^{-1}$)** | **Ref.** |
|---|---|---|---|---|
| | RP/C | 25-3000 | 760-78 | |
| **1** | BP-C (1:1) | 25-500 | 840-260 (420-130)[a] | [2] |
| **2** | S/O coped porous hard carbon | 50-1000 | 240-158 | [3] |
| **3** | BP-C | 50-500 | 367-90 | [4] |
| **4** | $Sn_3P_4$-C | 50-1000 | 340-200 | [5] |
| **5** | $Sn_3P_4$-N-doped CNFs | 50-2000 | 514-169 | [6] |
| **6** | N-doped CNFs | 100-5000 | 238-126 | [7] |
| **7** | P-O-doped graphene | 50-2000 | 450-160 | [8] |
| **8** | Hollow carbon | 28-560 | 320-100 | [9] |
| **9** | Carbon/Sn | 50-500 | 310-150 | [10] |
| **10** | N-rich carbon | 33.6-5040 | 250-154 | [11] |
| **11** | Graphite | 50-1500 | 250-10 | [12] |
| **12** | Soft carbon | 6.975-1395 | 273-140 | [13] |
| **13** | N-doped hierarchically porous carbon | 50-500 | 287-193 | [14] |
| **14** | N,P-codoped porous C sheets/CoP | 50-2000 | 174-54 | [15] |
| **15** | Red P@CN | 100-2000 | 696-323 | [16] |
| **16** | P@TBMC | 50-2000 | 484-136 | [17] |

[a] The specific capacity was calculated based on the total mass of P-C composites.

**Table S2.** Detailed data corresponding to Figure 5b

| | Configuration of full cell | Current density (mA $g^{-1}$) | Cycle number | Anode specific discharge capacity (mA h $g^{-1}$) | Ref. |
|---|---|---|---|---|---|
| | RP/C-KMnHCF | 150 | 50 | 401 | |
| | | 600 | 40 | 372 | |
| | | 1000 | 680 | 203 | |
| **1** | NCNF-650-KPB | 200 | 30 | 197 | [7] |
| **2** | HINCA-PTCDA | 140 | 20 | 180 | [9] |
| **3** | PNCM-PDCTA | 100 | 75 | 113 | [18] |
| | | 500 | 150 | 101 | |
| **4** | Bi-KFeHCF | 800 | 350 | 200 | [19] |

**Table S3.** Detailed data corresponding to Figure S21. The average discharge voltage was obtained from dividing the discharge energy (Wh) by discharge capacity (A h).

| | Configuration of full cell | Current density (mA $g^{-1}$) | Average discharge voltage (V) | Energy density (W h $kg^{-1}$) | Ref. |
|---|---|---|---|---|---|
| | RP/C-KMnHCF | 65 | ~3.0 | 193 ($2^{nd}$ cycle) | |
| | | 1000 | ~3.4 | 89 ($50^{th}$ cycle) | |
| | | 1000 | ~2.7 | 57 ($680^{th}$ cycle) | |
| **1** | NCNF-650-KPB | 200 | - | 130 | [7] |
| **2** | PNCM-PDCTA | 100 | - | 153.5 | [18] |
| **3** | Bi-KFeHCF | 200 | ~2.8 | 108.1 | [19] |

**Reference**

[1] L. Xue, Y. Li, H. Gao, W. Zhou, X. Lü, W. Kaveevivitchai, A. Manthiram, J. B. Goodenough, J. Am. Chem. Soc. **2017**, 139, 2164.
[2] I. Sultana, M. M. Rahman, T. Ramireddy, Y. Chen, A. M. Glushenkov, J. Mater. Chem. A **2017**, 5, 23506.
[3] M. Chen, W. Wang, X. Liang, S. Gong, J. Liu, Q. Wang, S. Guo, H. Yang, Adv. Energy Mater. **2018**, 1800171.
[4] X. Wu, W. Zhao, H. Wang, X. Qi, Z. Xing, Q. Zhuang, Z. Ju, J. Power Sources **2018**, 378, 460.
[5] W. Zhang, J. Mao, S. Li, Z. Chen, Z. Guo, J. Am. Chem. Soc. **2017**, 139, 3316.
[6] W. Zhang, W. K. Pang, V. Sencadas, Z. Guo, Joule **2018**, 8, 1534.
[7] Y. Xu, C. Zhang, M. Zhou, Q. Fu, C. Zhao, M. Wu, Y. Lei, Nat. Commun. **2018**, 9, 1720.
[8] G. Ma, K. Huang, J.-S. Ma, Z. Ju, Z. Xing, Q.-c. Zhuang, J. Mater. Chem. A **2017**, 5, 7854.
[9] D.-S. Bin, X.-J. Lin, Y.-G. Sun, Y.-S. Xu, K. Zhang, A.-M. Cao, L.-J. Wan, J. Am. Chem. Soc. **2018**, 140, 7127.
[10] K. Huang, Z. Xing, L. Wang, X. Wu, W. Zhao, X. Qi, H. Wang, Z. Ju, J. Mater. Chem. A **2018**, 6, 434.
[11] C. Chen, Z. Wang, B. Zhang, L. Miao, J. Cai, L. Peng, Y. Huang, J. Jiang, Y. Huang, L. Zhang, J. Xie, Energy Storage Materials **2017**, 8, 161.
[12] Z. Tai, Q. Zhang, Y. Liu, H. Liu, S. Dou, Carbon **2017**, 123, 54.
[13] Z. Jian, W. Luo, X. Ji, J. Am. Chem. Soc. **2015**, 137, 11566.
[14] X. Qi, K. Huang, X. Wu, W. Zhao, H. Wang, Q. Zhuang, Z. Ju, Carbon **2018**, 131, 79.
[15] J. Bai, B. Xi, H. Mao, Y. Lin, X. Ma, J. Feng, S. Xiong, Adv. Mater. **2018**, 1802310.
[16] D. Liu, X. Huang, D. Qu, D. Zheng, G. Wang, J. Harris, J. Si, T. Ding, J. Chen, D. Qu, Nano Energy **2018**, 52, 1.
[17] P. Xiong, P. Bai, S. Tu, M. Cheng, J. Zhang, J. Sun, Y. Xu, Small **2018**, 14, 1802140.
[18] Y. Xie, Y. Chen, L. Liu, P. Tao, M. Fan, N. Xu, X. Shen, C. Yan, Adv. Mater. **2017**, 29, 1702268.
[19] K. Lei, C. Wang, L. Liu, Y. Luo, C. Mu, F. Li, J. Chen, Angew. Chem. **2018**, 130, 4777.
